# Error Correction and the Structure of Inter-Trial Fluctuations in a Redundant Movement Task

**DOI:** 10.1371/journal.pcbi.1005118

**Published:** 2016-09-19

**Authors:** Joby John, Jonathan B. Dingwell, Joseph P. Cusumano

**Affiliations:** 1 Department of Engineering Science and Mechanics, The Pennsylvania State University, University Park, Pennsylvania, United States of America; 2 Department of Kinesiology and Health Education, The University of Texas at Austin, Austin, Texas, United States of America; Western University, CANADA

## Abstract

We study inter-trial movement fluctuations exhibited by human participants during the repeated execution of a virtual shuffleboard task. Focusing on skilled performance, theoretical analysis of a previously-developed general model of inter-trial error correction is used to predict the temporal and geometric structure of variability near a goal equivalent manifold (GEM). The theory also predicts that the goal-level error scales linearly with intrinsic body-level noise via the *total body-goal sensitivity*, a new derived quantity that illustrates how task performance arises from the interaction of active error correction and passive sensitivity properties along the GEM. Linear models estimated from observed fluctuations, together with a novel application of bootstrapping to the estimation of dynamical and correlation properties of the inter-trial dynamics, are used to experimentally confirm all predictions, thus validating our model. In addition, we show that, unlike “static” variability analyses, our dynamical approach yields results that are independent of the coordinates used to measure task execution and, in so doing, provides a new set of task coordinates that are intrinsic to the error-regulation process itself.

## Author Summary

During the repeated execution of precision movement tasks, humans face two formidable challenges from the motor system itself: dimensionality and noise. Human motor performance involves biomechanical, neuromotor, and perceptual degrees of freedom far in excess of those theoretically needed to prescribe typical goal-directed tasks. At the same time, noise is present in the human body across multiple scales of observation. This high-dimensional and stochastic character of biological movement is the fundamental source of variability ubiquitously observed during task execution. However, it is becoming clear that these two challenges are not merely impediments to be overcome, but rather hold a key to understanding how humans maintain motor performance under changing circumstances, such as those caused by fatigue, injury, or aging. In this work, by studying skilled human participants as they play a virtual shuffleboard game, we demonstrate the fundamental importance of adopting a dynamical perspective when analyzing the motor variability observed over many trials. Using this dynamical approach, we can not only study the geometry of observed inter-trial variability, but can also theoretically describe and experimentally characterize how it is temporally generated and regulated. Furthermore, our theoretical framework and model-based data analysis approach helps to unify previous variability analysis approaches based on stability, correlation, control theory, or task manifolds alone. This conceptual unification supports the idea that such seemingly disparate features of motor variability arise from a single, relatively simple underlying neurophysiological process of motor regulation.

## Introduction

During the repeated execution of goal-directed movements, statistical variability is always observed from one trial to the next, and this motor variability has long been a major focus of movement neuroscience [1–3]. It is generally believed that these inter-trial fluctuations contain crucial information about how the neuromotor system organizes itself to meet task requirements in the face of physical constraints, external perturbations, and motor noise [4–9]. Indeed, there is increasing evidence that inherent biological noise, which is present at multiple scales from the level of motor units down to the level of genes, may play a crucial physiological function in the nervous system [7, 10, 11]. However, the process by which this multiscale noise comes to be expressed as variability at the organismic level is still far from completely understood.

There is an excess of body-level degrees of freedom over those needed to specify the outcome of a typical goal-directed movement, and it is natural to expect this redundancy to affect the structure of observed variability. A number of data analysis approaches [12–14] have been developed to examine the effect of this redundancy using *task manifolds*, which are surfaces in a suitably-defined space of biomechanical observables, or “body states” (e.g., joint kinematic variables), that contains all possible task solutions. By definition, every point in a task manifold corresponds to a body state that results in perfect task execution, and so, as a consequence, only body-level deviations away from the manifold result in error at the goal level.

Originally inspired by ideas from research in redundant robotics, uncontrolled manifold (UCM) analysis [12, 15–17] assumes that the task manifold is defined at each instant along a given movement trajectory, and in typical applications takes the task’s goal to be represented by the average movement in a time-normalized set of trials. The ratios of normalized variances orthogonal and tangent to a candidate manifold are then used to identify possible “control variables”, with the expectation that there should be a larger variance along the manifold than normal to it. In a similar vein, motor learning has been studied by statistically decomposing observed body-level variability into tolerance, noise, and covariation (TNC) empirical “costs”, [13, 18–20], all three of which are defined with respect to a task manifold. In contrast with UCM analysis, the TNC approach conceives of the task manifold as existing in a minimal space of variables needed to specify task execution (e.g., the position and velocity of a ball at release when throwing at a target). In addition to using its covariation cost to characterize the alignment of body-level variability with the task manifold, TNC analysis crucially relates the goal-level variability to error at the body level via its tolerance cost.

This relationship between body and goal-level variability was the initial focus of a sensitivity analysis method based on the goal equivalent manifold (GEM) concept [14]. Like TNC, the GEM analysis defines its task manifold using only a minimal set of variables needed for task specification, however it makes direct use of an explicit *goal function* that serves as a hypothesis on the task strategy being used. The zeros of the goal function give body states yielding perfect task execution, and the set of all such solutions then gives the GEM. In addition to defining the GEM, the goal function provides a theoretical definition of the “passive” sensitivity (i.e., sensitivity independent of any applied control) to body-level disturbances, via the singular values of the goal function’s Jacobian matrix [14, 21].

While the initial GEM-based sensitivity analysis was useful for describing the geometrical structure of observed variability and quantifying motor performance, like the UCM and TNC approaches it did not provide an analysis of the *temporal* structure of observed inter-trial fluctuations. This limitation was addressed by subsequent developments that incorporated optimal control ideas with the GEM to create a dynamical, model-based data analysis framework. Optimal control in the presence of redundancy has been proposed as a theoretical basis for models of the neuromotor system [22, 23], and the minimum intervention principle (MIP) [23, 24] posits that little or no control will be exerted along the task manifold, since to do so would entail a waste of control effort. The expanded GEM data analysis framework allows one to create theoretical models of inter-trial fluctuations that can be used for hypothesis testing against movement data from human participants [25–27].

This initial work has demonstrated the central importance of taking a dynamical approach when analyzing motor variability. A fundamental feature of variability highlighted by these studies is that inter-trial fluctuations are found to be *dynamically* anisotropic with respect to the GEM [25–29]: that is, it is found that the local stability and correlation properties are congruent with the local GEM geometry, with greater stability and lower temporal correlation being associated with the components of time series transverse to the GEM, and lower stability and greater correlation for times series components along the GEM. A similar directionality in correlation properties has been found in a study of skill acquisition [30]. However, such studies have tended to examine these dynamical properties in isolation, and it is not completely clear how the various temporal properties (e.g., local stability multipliers, lag-1 correlations, etc.) relate, if at all, to the purely geometrical features of inter-trial variability arising from the task manifold itself (e.g. variance ratios, passive sensitivity). In particular, it remains an open question whether these various features of inter-trial variability should be considered as manifestations of unique neurophysiological phenomena each in their own right, or if, conversely, they are epiphenomena that naturally arise from a single, underlying regulatory process. In this paper we present evidence that supports the latter, more parsimonious interpretation.

To this end, we examine the performance of human participants as they play a virtual shuffleboard game. We chose shuffleboard for this study because it is among the simplest tasks exhibiting task-level redundancy, and is thus both mathematically and experimentally tractable. As such, it serves as a “model problem” for a much broader class of goal-directed tasks which can be expected to exhibit similar variability characteristics. Observed inter-trial fluctuations are modeled as the output of the perception-action system as participants attempt to hit the target in each trial by correcting error in the previous trial. We focus on skilled performance, and, starting with a previously-developed general model for inter-trial error correction [21, 26, 28], we present a theoretical analysis using the shuffleboard task as an illustrative example. The analysis yields theoretical predictions about the geometrical and temporal structure of inter-trial variability, culminating in a prediction of how GEM geometry, passive sensitivity, and active error correction combine to yield task performance. Specifically, we show that the scaling of the root mean square (RMS) error at the target is determined by the *total body-goal sensitivity*, which is, in effect, a total “gain” mapping body-level fluctuations to the goal level.

We also address a critical technical issue that arises when experimentally testing our theoretical predictions. For skilled performance, the local geometric stability properties of the fluctuations play a fundamental role, with such properties being determined theoretically by an eigenanalysis of a linearized model. Unfortunately, numerical estimates of eigenvalues and eigenvectors are known to be highly sensitive to errors in the matrix estimate [31], which are themselves unavoidable when the matrix is found using regression on experimental data. This problem is compounded by the relatively small data sets available in typical human subjects experiments. In this paper we present a new method for estimating all of our dynamical quantities based on bootstrapping [32–34], which allows us to estimate the complete underlying probability distribution for each quantity considered, resulting in the most robust demonstration to date of the degree to which dynamical anisotropy is present in inter-trial movement data. Furthermore, this data analysis allows us to confirm the theoretical performance scaling prediction to high precision, not only showing how the individual participants performed in this particular task, but also validating the many assumptions underlying our theoretical derivation.

Studies of variability using task solution manifolds typically assume that they are embedded in a space of variables with identical physical dimension, such as, for example, joint angles [14, 15, 35], muscle activation [36, 37], or finger forces [16, 38, 39]. Such situations have tended to obscure a fundamental difficulty if one intends to make inferences based on the relative magnitude of fluctuations normal and tangent to any hypothesized manifold: namely, that multivariate statistics are not invariant under coordinate transformations. This issue was recently recognized in the context of movement variability analysis [30, 40], but is a well-known problem in multivariate statistics. Indeed, the widespread utility of principal component analysis [41, 42] is based in part on the fact that correlations between variables can be completely removed with properly selected linear coordinate transformations.

It is clearly highly desirable that the inferences we make about the motor system be invariant under coordinate transformations. While it is possible to normalize the variables and make the data dimensionless, such an approach does not completely resolve the scaling issue because the choice of the normalizing constant is, in most cases, arbitrary. This problem becomes even more acute when the task manifold resides in a space composed of different physical quantities, for example positions and velocities. Given the central role played by local geometric stability in our approach, we are able to exploit the well-known fact that such dynamical properties are invariants that do not depend on the coordinates used [43, 44]. We therefore show that our approach provides a coordinate-independent characterization of the variability observed in our experiments, suggesting that the local geometric stability analysis of inter-trial fluctuations provides a new set of task coordinates that are intrinsic to the error regulation process itself.

## Methods

This section begins with a discussion of the key concepts and models that theoretically ground our approach, and that culminate in a set of four experimental hypotheses. With this theoretical background as foundation, we then describe our experimental virtual shuffleboard game, the experimental protocol, and our data analysis methods.

### Ethics Statement

All participants provided informed consent, as approved by the Institutional Review Board at The Pennsylvania State University.

### The Shuffleboard Task and GEM

**Fig 1:**
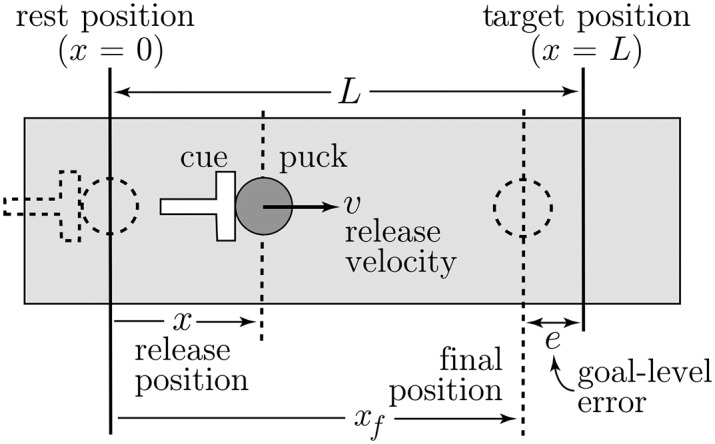
Schematic of a shuffleboard task: the shuffleboard cue pushes the puck from rest and releases it at a position *x* with a velocity *v* when the contact force between puck and cue decreases to zero. Thereafter, the puck decelerates due to the Coulomb friction force between the puck and the board, and eventually comes to rest at *x*_*f*_. The target is at a distance *L* from the initial position and the goal-level error is *e* = *x*_*f*_ − *L*.

Fig 1 shows a schematic of a theoretical shuffleboard game. The entire game takes place along a straight line. Starting the puck at *x* = 0, the shuffleboard cue is accelerated from rest while in contact with the puck. Thereafter, the cue decelerates and, when the contact force between it and the puck reaches zero, the puck is released with position and velocity *x* and *v*, respectively. Once released, the puck slides on the board and is decelerated by the force of Coulomb friction, with kinetic coefficient *μ*, between the board and the puck. The puck eventually comes to rest at *x* = *x*_*f*_. The goal-level error, *e* = *x*_*f*_ − *L*, is the distance between the final puck position and the target.

Elementary Newtonian mechanics gives the equation of motion for the puck after release as $\ddot{x}=-\mu g$, where *g* is the gravitational acceleration constant. For arbitrary initial conditions *x* and *v* just after release, and final velocity *v*_*f*_ = 0, the equation of motion is easily integrated to give −*v*^2^ = −2*μg*(*x*_*f*_ − *x*). Since perfect execution (hitting the target) requires *e* = *x*_*f*_ − *L* = 0, we then obtain a goal function for the task as

$$e=f\left(x,v\right)=v^{2}+2\mu g\left(x-L\right)\;.$$ (1)

Any values of *x* and *v* for which *e* = *f*(*x*, *v*) = 0 result in perfect task execution (zero error at the goal level).

Dimensionless quantities $\tilde{x}=x/R$, $\tilde{v}=v/\sqrt{2gR}$, and $\tilde{L}=L/R$ can be defined for some length scale *R*. Note that the exact value of *R* used in this rescaling has no significant bearing on our results: it was chosen for convenience so that when plotting experimental data the rescaled release position $\tilde{x}=x/R\approx 1$. For the experiments described in what follows, we took *L* = 200cm and *R* = 20cm, so that the target was located at a distance of $\tilde{L}=10$ dimensionless units. Using these rescalings in Eq (1) gives, after rearranging and *dropping tildes*, the goal function in *dimensionless* form as

$$f\left(x,v\right)=\frac{v^{2}}{\mu }+x-10\;.$$ (2)

Henceforth we use the dimensionless goal function of Eq (2).

**Fig 2:**
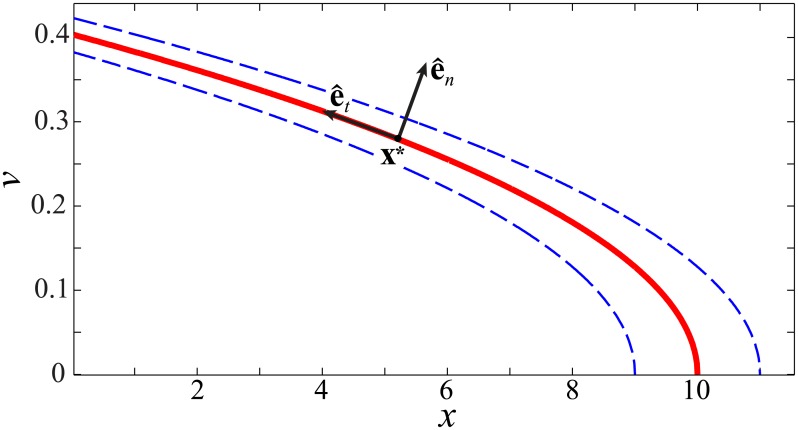
Typical GEM (solid curve) for the shuffleboard task, obtained as zeros of the goal function Eq (2), plotted in the dimensionless (*x*, *v*) body state space. Dashed curves indicate ±10% constant error contours at the goal (as a percentage of distance to the goal). For this particular plot, *μ* ≈ 0.016. Also shown are the unit vectors tangent and normal to the GEM, ${\hat{e}}_{t}$ and ${\hat{e}}_{n}$, near a representative operating point x* (Eqs (5) and (6)): small deviations along ${\hat{e}}_{t}$ do not cause error at the target (i.e., they are *goal equivalent*), while deviations along ${\hat{e}}_{n}$ do (i.e., they are *goal relevant*). Note that the distance between contours increases from left to right, indicating a decrease in passive sensitivity (see Eq 8) along the GEM.

There are an infinite number of states (*x*, *v*) that are zeros to Eq (2), corresponding to trials that hit the target perfectly. In this simple case, we can solve for this set analytically, and find, as shown in Fig 2, that it forms a 1D goal equivalent manifold (GEM)

$$G=\left\{\;\left(x,v\right)\;\;|\;\;v^{2}=\mu \left(10-x\right)\;\right\}\;,$$ (3)

which has the shape of a parabola in the (*x*, *v*) plane. Since the performance is completely determined by the values of *x* and *v* at release, we take as our body state **x** = (*x*, *v*)^T^ (where the superscript T denotes the transpose). Note that the goal function *f*(**x**) ≠ 0 for “strategies” **x** that are not exactly on the GEM: for this task, this value is identical to the goal-level error, *e*.

The GEM represented in Fig 2 exists independently of who or what performs the task. Actuating the shuffleboard cue with a single degree of freedom pneumatic actuator, a robot with tens of degrees of freedom, or a biological organism with thousands of degrees of freedom does not affect the requirements in the (*x*, *v*) body state space needed to hit the target. Furthermore, the GEM has been defined without any consideration of the control that might be applied to correct errors from one trial to the next: even a completely uncontrolled system that randomly assigned values of *x* and *v* for each trial would have the same GEM.

For a skilled participant whose performance is perfect *on average*, we assume that the state will be near the GEM and write **x** = x* + **u**, where the operating point $x^{*}={\left(x^{*},v^{*}\right)}^{T}\in G$ represents the average perfect trial on the GEM, and **u** = (*p*, *q*)^T^ is a small fluctuation. Substitution into the goal function Eq (2) and linearizing about **u** = (0, 0)^T^ then gives

$$e\;=\;\frac{{\left(v^{*}+q\right)}^{2}}{\mu }+\left(x^{*}+p\right)-10\;\approx \;\left(1\;\;\frac{2v^{*}}{\mu }\right)\left(\begin{array}{c} p\\ q \end{array}\right)\;≜\;Au\;,$$ (4)

where $A=(\frac{\partial f}{\partial x}\;\frac{\partial f}{\partial v})$, with derivatives evaluated at (x*, v*), is the 1 × 2 body-goal variability matrix [14] that maps body-level perturbations **u** into goal-level error *e*.

The null space N of A, defined by N={u|Au=0}, contains fluctuations that are *goal equivalent*, i.e., that to leading order have no effect on the goal level error. Using this definition, the unit tangent vector to the GEM is found to be

$${\hat{e}}_{t}=\frac{1}{\sqrt{1+{\left(\frac{2v^{*}}{\mu }\right)}^{2}}}\left(\begin{array}{c} -\frac{2v^{*}}{\mu }\\ 1 \end{array}\right),$$ (5)

giving the 1D goal-equivalent subspace as $N=\mathrm{span}\{{\hat{e}}_{t}\}$, which is also the subspace tangent to the GEM at x* (again, see Fig 2). In contrast, the row space R of A contains fluctuations that result in error at the goal and, hence, are *goal relevant*. This 1D space is orthogonal to the GEM, so that $R=\mathrm{span}\{{\hat{e}}_{n}\}$, where ${\hat{e}}_{n}$ is the unit normal to the GEM given by

$${\hat{e}}_{n}=\frac{1}{\sqrt{1+{\left(\frac{2v^{*}}{\mu }\right)}^{2}}}\left(\begin{array}{c} 1\\ \frac{2v^{*}}{\mu } \end{array}\right).$$ (6)

Given a fluctuation **u** from the operating point x*, its goal-relevant and goal-equivalent components are found using the inner products

$$u_{R}=u\cdot {\hat{e}}_{n}\;\text{and}\;u_{N}=u\cdot {\hat{e}}_{t}\;,$$ (7)

respectively. Using these, one can readily compute from observations the sample standard deviations of goal-relevant and goal-equivalent fluctuations, $\sigma_{R}$ and $\sigma_{N}$, respectively.

The singular values of the body-goal matrix A determine how fluctuations **u** get amplified onto the target [14], and so determine the *sensitivity* of the performance to body-level errors. Since the sensitivity depends only on the goal function, it is independent of any specific inter-trial control mechanism, and so is considered to be a *passive* property of the task. For the shuffleboard game, A has one singular value *s*, which is given by [31]

$$s=\sqrt{1+{\left(\frac{2v^{*}}{\mu }\right)}^{2}}\;.$$ (8)

Thus, the passive sensitivity is a function of the friction coefficient, *μ*, and the speed at the operating point, v*, with the latter indicating that *s* is not constant along the GEM. Given *s*, Eq (4) can then be used to obtain the RMS goal-level error as

$$\sigma_{e}=s\sigma_{R}\;,$$ (9)

which is a special case of the general expression obtained in [14]. Thus, the passive sensitivity “explains” the goal level error, but only when the goal-relevant fluctuations are taken as given. However, the scale of those fluctuations, $\sigma_{R}$, is itself determined by the active process of inter-trial error correction.

### Modeling Inter-Trial Fluctuations

As discussed previously, the GEM and body-goal sensitivity are passive properties of the task that exist prior to the imposition of any error-correcting control. Here, we “close the loop” on the problem by discussing simple perception-action models of inter-trial error correction. For clarity, we present our modeling framework with a bit more generality than will ultimately be needed. Additional background and details can be found in [26, 28].

A typical experiment for a goal-directed task with *N* trials results in a time series of the body state variable, ${\left\{x_{k}\right\}}_{k=1}^{N}$, and a corresponding time series of goal-level errors, ${\left\{e_{k}\right\}}_{k=1}^{N}$. We consider these time series to result from the process of error-correction used by participants as they make adjustments after each trial, and model the fluctuation dynamics with update equations of the form [21, 26, 28]:

$$x_{k+1}=x_{k}+G\left(I+N_{k}\right)c\left(x_{k}\right)+\nu_{k}\;,$$ (10)

in which: **c**(**x**_*k*_) is an inter-trial, error-correcting controller depending on the current state; N_*k*_ is a matrix representing signal-dependent noise in the motor outputs [45]; and ***ν***_*k*_ is an additive noise vector representing unmodeled effects from perceptual and neuromotor sources. The diagonal matrix of gains, G, is included as a convenient way to detune the model away from optimality when **c** is an optimal controller designed initially with G = I [26].

Error-correcting models with mathematical form similar to Eq (10) have been used to study motor learning [46–48] and to understand the effect of motor noise. These previous efforts have not focused on the role of task level redundancy, or attempted to relate body-level fluctuations to those at some external goal, as we do here. However, in contrast to these previous studies, we do not make reference to hidden internal state variables related, for example, to motor planning, but instead construct our models at the level of experimentally-observable task-relevant kinematic variables. As a consequence, our models cannot be used to disambiguate the effect of noise due to motor planning from that due to motor execution [46]. Our focus here is not on how internal “neuronal” state variables are dynamically mapped to kinematic output variables, but rather how the body-level task variables are mapped onto the goal-level task error in the presence of redundancy. Hence, our study takes place at a different level of description than studies aimed at understanding the physiological origin of motor noise and its role in motor learning. Models with the general form of Eq (10) can be viewed as the *between*-trial component of a hierarchical motor regulation scheme that makes error-correcting adjustments to an approximately “feed forward,” *within*-trial component.

Focusing once again on skilled movements, we write **x**_*k*_ = x* + **u**_*k*_ as was done leading up to Eq (4), where **u**_*k*_ are small perturbations from the operating point x*. Assuming, in addition, small noise terms N_*k*_ and ***ν***_*k*_, we can linearize the controller Eq (10) [21, 28] about **u**_*k*_ = 0 to obtain:

$$u_{k+1}=Bu_{k}+\nu_{k}\;,$$ (11)

where the matrix B = I+GJ, and J = ∂**c**/∂**x** is the Jacobian of the controller evaluated at x*. Note that, to leading order, signal dependent noise does not affect the inter-trial dynamics near the GEM [28]. Thus, small fluctuations are governed by the linear map of Eq (11), and the eigenvalues and eigenvectors of B determine the local dynamic stability properties of the system [44, 49, 50]. Specifically, eigenvalues *λ* with magnitude near zero (|*λ*|≈0) indicate that deviations from the GEM are rapidly corrected, whereas positive eigenvalues strictly less than but closer to one (0 ≪ *λ* < 1) indicate that deviations are only weakly corrected (that is, they are allowed to “persist”). Note that values of *λ* > 1 indicate instability, indicating that deviations would continue to grow in successive trials, something that is not expected in experiments. For the shuffleboard task, the body states are 2-dimensional, so that B is a 2 × 2 matrix possessing two eigenvalues, {*λ*_*w*_, *λ*_*s*_}, and two eigenvectors, $\left\{{\hat{e}}_{w},{\hat{e}}_{s}\right\}$, where the subscripts *w* and *s* indicate weakly and strongly stable directions, as described below. We limit our discussion to the case of real, distinct eigenvalues, which has been found to be sufficient in experimental applications to date.

In [26], **c** was found analytically as an optimal controller using different specified cost functions. Because goal-level error was minimized as a cost, the goal function (which, for the current paper, is given by Eq 2) was built into the model, and so the effect of the GEM was explicitly included. In studies of this type, the model is used to generate simulated data, which is then statistically compared to experimental data to “reverse engineer” the controller used by human participants. Furthermore, if one wishes to study local stability properties via Eq (11), the matrix B can, in principle, be obtained analytically by differentiation.

In contrast, in this work we take a simpler, empirical approach: instead of formulating an explicit optimal controller, linear regression is used to estimate the matrix B of Eq (11) directly from the experimental fluctuation data. The eigenstructure of the estimated B is then obtained and compared to the geometry of the shuffleboard GEM (Fig 2). Thus, other than the assumption of closeness to an operating point $x^{*}\in G$ (i.e., of linearity), the controller is not assumed to to be optimal, nor is the GEM encoded into it in any way. Thus, any structure in the data related to the presence of the GEM is a property of the observed fluctuation dynamics: it has not been imposed by the model.

### Relating Fluctuations at Body and Goal Levels

Task manifold methods applied to a variety of motor tasks have shown that the body-level variability observed during skilled task execution will tend to have greater variance along the task manifold than normal to it. Indeed, anisotropy in the variability is typically taken to demonstrate that a hypothesized task manifold is being used to organize motor control [12, 16]. Such results are consistent with a generalized interpretation of the UCM hypothesis and the MIP: namely, that while disturbances along the task manifold are not truly “uncontrolled”, they are, at least, more *weakly* controlled than those normal to it. However, movement variability may be “structured” (i.e., may exhibit anisotropy) for biomechanical and/or neurophysiological reasons that are unrelated to control [36]. In addition, variance-based analyses are vulnerable to ambiguities related to the coordinate dependence of variability statistics [28, 40], and by themselves do not provide any insight into how observed fluctuations are dynamically generated and regulated [28, 51].

A number of researchers have addressed this last limitation by combining task manifold ideas with time series analysis of statistical persistence [25–27, 30, 51–54], as measured either via detrended fluctuation analysis (DFA) [55, 56] or autocorrelations. Generally speaking, a time series exhibits statistical persistence if, given fluctuations in one direction, subsequent fluctuations are likely to be in the same direction. If subsequent fluctuations are likely to be in the opposite direction, the time series is said to be antipersistent, and if subsequent fluctuations are equally likely to be in either direction the time series is non-persistent or, alternatively, uncorrelated. As was shown in [25], the coherent interpretation of persistence results requires the consideration of error-correcting control near the task manifold: there is greater statistical persistence along the manifold, where the control is weak, than perpendicular to it, where the control is strong. These types of results are, again, consistent with a generalized interpretation of the MIP [28].

All of the above-cited studies lead us to expect *dynamical* anisotropy in inter-trial fluctuations. That is, the temporal structure of fluctuations should reflect the operation of a controller that strongly acts against goal-relevant deviations by pushing subsequent body-states toward the GEM, while only weakly acting to correct goal-equivalent deviations along the GEM.

**Fig 3:**
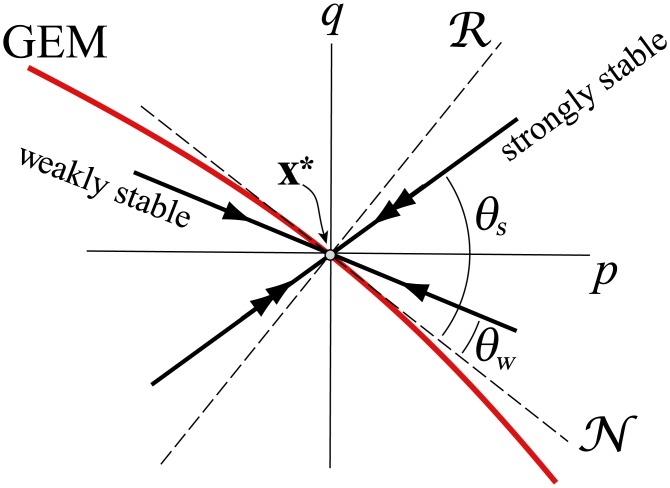
Schematic showing the goal-equivalent (null) space N and goal-relevant (column) space R of fluctuations about an operating point x* on the GEM, and the relative orientation of the weakly (single arrow) and strongly (double arrow) stable subspaces determined by the eigenvectors of a 2 × 2 matrix B (Eq (11)), as given by angles *θ*_*w*_ and *θ*_*s*_, respectively. Also shown are the coordinate axes of the position and velocity fluctuations, *p* and *q*, respectively. Note that *θ*_*w*_ is exaggerated for clarity: we expect *θ*_*w*_ ≈ 0. The strongly stable direction is transverse, but not necessarily perpendicular, to the GEM.

Since in this paper we focus on skilled movements, we make direct use of the linearized model Eq (11). For an ideal MIP controller, the complete absence of control along the GEM would result in neutral stability along it, as well, meaning that one eigenvector of the matrix B (Eq (11)) would be identical to the unit tangent ${\hat{e}}_{t}$, and its associated eigenvalue would be *λ* = 1. However, such a scenario in the presence of motor noise would result in an unbounded random walk along the GEM, something which has yet to be observed in experiments. Thus, we expect the inter-trial dynamics to be slightly perturbed from what one would expect for a perfect MIP controller, giving one *weakly stable* eigenvalue less than, but somewhat close to, 1 (i.e., 0 ≪ *λ*_*w*_ < 1) with an associated unit eigenvector **e**_*w*_ that is close to ${\hat{e}}_{t}$, but slightly rotated. In contrast, the *strongly stable* eigenvalue, *λ*_*s*_, indicates vigorous correction of deviations off of the GEM, so that |*λ*_*s*_|≈0 and **e**_*s*_ is transverse (but not necessarily perpendicular) to the GEM. The general geometry of the situation, in which local stability properties are overlaid on the GEM near an operating point x*, is show schematically in Fig 3.

The fluctuations **u**_*k*_ in the original, laboratory coordinates (e.g., representing speed and position for the shuffleboard game) can be transformed into new fluctuations expressed in eigencoordinates via the linear coordinate transformation

$$u_{k}=Ez_{k}\;,$$ (12)

where E is the matrix containing ${\hat{e}}_{w}$ and ${\hat{e}}_{s}$ as its columns. Note that E is not typically an orthogonal matrix because the eigenvectors of B are not usually perpendicular. Using this transformation, Eq (11) becomes

$$z_{k+1}=E^{-1}BEz_{k}+E^{-1}\nu_{k}≜Qz_{k}+n_{k}\;.$$ (13)

where **z** = (*z*_*w*_, *z*_*s*_)^T^ are the fluctuations expressed in weak-strong eigencoordinates, the diagonal matrix Q = E^−1^ BE has *λ*_*w*_ and *λ*_*s*_ along its diagonal, and **n** = (*n*_*w*_, *n*_*s*_)^T^ is the transformed additive noise term. That is, the transformation Eq (12) decouples the dynamics in the weak and strong directions so that Eq (13) can be written as

$$z_{w,k+1}=\lambda_{w}\;z_{w,k}+n_{w,k}$$ (14)

$$z_{s,k+1}=\lambda_{s}\;z_{s,k}+n_{s,k},$$ (15)

in which *z*_*w*, *k*_ and *z*_*s*, *k*_ are simply the components of **z**_*k*_ in the weak and strong directions, respectively. This “diagonalized” form of the system illustrates the action of each eigenvalue on fluctuations in their respective directions: in the absence of noise an eigenvalue close to zero will eliminate a given fluctuation on the very next trial, whereas a positive eigenvalue a bit less than 1 will allow fluctuations to persist over many trials. The decomposition of Eqs (14) and (15) is intrinsic to the fluctuation dynamics created by inter-trial error correction, and so differs significantly from “static” decompositions using, for example, the normal and tangent to the GEM, or principal component analysis [42].

From Eq (7) and the transformation Eq (12) we can relate the standard deviations of fluctuations in the goal-relevant and strongly-stable directions as

$$u_{R}={\hat{e}}_{n}\cdot u={\hat{e}}_{n}\cdot \left(z_{w}{\hat{e}}_{w}+z_{s}{\hat{e}}_{s}\right)\approx \beta z_{s}\;⟹\;\sigma_{R}\approx \beta \sigma_{s}\;,$$ (16)

where $\beta \;≜\;{\hat{e}}_{n}\;\cdot {\hat{e}}_{s}=\sin\left(\theta_{s}\right)$ (see Fig 3) and we have assumed, consistent with a generalized MIP, that the weakly stable direction is nearly tangent to the GEM, so that ${\hat{e}}_{w}\approx {\hat{e}}_{t}\Rightarrow {\hat{e}}_{n}\;\cdot {\hat{e}}_{w}\approx 0$. Squaring both sides of Eq (14), taking the ensemble average (as indicated by angle brackets), and assuming that the noise and fluctuations at trial *k* are uncorrelated, yields

$$\left\langle z_{w,k+1}^{2}\right\rangle =\lambda_{w}^{2}2mu\left\langle z_{w,k}^{2}\right\rangle +\left\langle n_{w,k}^{2}\right\rangle \;⟹\;\sigma_{w}=\frac{\sigma_{nw}}{\sqrt{1-\lambda_{w}^{2}}}\;,$$ (17)

where $\sigma_{nw}^{2}\equiv \langle n_{w,k}^{2}\rangle$, and in which we have used the fact that at steady state $\langle z_{w,k+1}^{2}\rangle =\langle z_{w,k}^{2}\rangle \equiv \sigma_{w}^{2}$. A similar calculation with Eq (15) gives

$$\sigma_{s}=\frac{\sigma_{ns}}{\sqrt{1-\lambda_{s}^{2}}}\;.$$ (18)

Eqs (17) and (18) show that as the eigenvalues approach 0, the “output” variance of the fluctuations approaches a minimum value equal to the variance of the “input” noise. Conversely, as the eigenvalues approach the stability boundary of 1, the output variance becomes unbounded (i.e., the fluctuations approach the behavior of a random walk).

Finally, substituting from Eq (16) into Eq (9), using Eq (18), and rearranging we find

$$\frac{\sigma_{e}}{\sigma_{ns}}\approx \frac{\beta s}{\sqrt{1-\lambda_{s}^{2}}}\;≜\;s_{\text{TOT}}\;,$$ (19)

where *s*_TOT_ is the *total body-goal sensitivity*, which quantifies how much intrinsic body-level fluctuations are amplified at the goal level. Note that *s*_TOT_ results from the interaction of the passive sensitivity (via *s*), the local GEM geometry (via *β* = sin*θ*_*s*_) and active control “strength” (via *λ*_*s*_).

### Statistical Persistence

Given *z*_*w*_ and *z*_*s*_ time series from the diagonalized controller of Eqs (14) and (15), we can compute the normalized lag-1 autocorrelations of the fluctuations in the weak and strong directions as

$$R_{w}\left(1\right)=\frac{\left\langle \left(z_{w,k+1}\right)\left(z_{w,k}\right)\right\rangle }{\sigma_{w}^{2}}\;\text{and}\;R_{s}\left(1\right)=\frac{\left\langle \left(z_{s,k+1}\right)\left(z_{s,k}\right)\right\rangle }{\sigma_{s}^{2}}\;,$$ (20)

respectively. This provides a simple quantification for the statistical persistence in both directions. However, multiplying Eq (14) by *z*_*w*, *k*_, taking the ensemble average, and assuming the additive noise is uncorrelated with the fluctuations so that 〈(*z*_*w*, *k*_)(*n*_*w*, *k*_)〉 = 0 gives

$$\left\langle \left(z_{w,k+1}\right)\left(z_{w,k}\right)\right\rangle =\lambda_{w}\left\langle \left(z_{w,k}\right)\left(z_{w,k}\right)\right\rangle \equiv \lambda_{w}\sigma_{w}^{2}\;.$$ (21)

Solving for *λ*_*w*_ in the above and comparing it to the definition *R*_*w*_(1) in Eq (20), we see that *R*_*w*_(1) ≡ *λ*_*w*_. Likewise, a similar calculation with Eq (15) shows *R*_*s*_(1) ≡ *λ*_*s*_. Thus, as a persistence measure the normalized lag-1 autocorrelation does not, theoretically speaking, provide information distinct from the eigenvalues *λ*_*w*_ and *λ*_*s*_. We include it here to demonstrate the connection between stability and this simple persistence measure. We use it later, as well, to serve as a consistency check on our experimental eigenvalue estimates.

To test for statistical persistence with a method independent from the eigenanalysis, one can apply detrended fluctuation analysis (DFA) [55, 56] with linear detrending to the *z*_*w*_ and *z*_*s*_ time series. The DFA algorithm yields a positive exponent, *α*, where *α* < 0.5 indicates antipersistence in a time series, *α* > 0.5 indicates persistence and *α* = 0.5 indicates non-persistence. Contrary to its most common use in the literature, in this work we *are not* using DFA to claim that observed fluctuations exhibit *long-range* persistence, but instead employ *α* merely as a convenient overall measure of persistence that, unlike the autocorrelation, does not require consideration of specific lags. Additional discussion regarding the application of DFA to movement variability data can be found in [28], including a review of its vulnerability to false positives when testing for long-range persistence [57–59].

### Coordinate Invariance

In this subsection we show how the dynamical analysis of inter-trial fluctuations allows us to characterize observed variability in a way that is insensitive to the choice of coordinates. Starting with some original body state variable **x**, consider a new variable **y** of the same dimension as **x**, with each being related by a general differentiable, invertible coordinate transformation **x** = **g**(**y**). Thus, the operating point expressed for each choice of coordinates is related by x* = **g**(y*), and we find that small fluctuations are related to lowest order by a linear transformation from:

$$x^{*}+u_{k}=g\left(y^{*}+v_{k}\right)\approx g\left(y^{*}\right)+Tv_{k}\;⟹\;u_{k}=Tv_{k}\;,$$ (22)

where **u**_*k*_ and **v**_*k*_ are the fluctuations expressed in terms of the old and new coordinates, respectively, and T is the square Jacobian matrix of the transformation **g** evaluated at y*.

Using Eq (22) to substitute for **u**_*k*_ into the linearized controller Eq (11) then gives, in a manner analogous to that used to obtain Eq (13):

$$v_{k+1}=T^{-1}BTv_{k}+T^{-1}\nu_{k}\;.$$ (23)

Clearly, the matrix T^−1^BT on the right-hand side of the above equation is congruent to the original B, and so will have the same eigenvalues, and, hence, the same stability properties.

As discussed in [28], the GEM itself is transformed when using the new coordinates. Recall from the discussion prior to Eq (5) that the tangent to the GEM is determined from the null space of the Jacobian to the goal function, A. That is, to leading order the fluctuation **u**_*k*_ is on the GEM whenever A**u**_*k*_ = 0. However, again using the transformation Eq (22), we see that A**u**_*k*_ = AT**v**_*k*_, showing that whenever **u**_*k*_ is on the GEM expressed in terms of the original coordinates, **v**_*k*_ is on the GEM expressed using the new coordinates. Thus, not only are the stability properties unaffected by coordinate transformations, the eigenvectors and GEM are transformed in a predictable way that preserves the topology near the operating point: that is, while changing coordinates will typically rotate and shear the picture somewhat, the overall arrangement illustrated in Fig 3 is preserved.

### Experimental Hypotheses

Following the above discussion, we are led to the following four theoretical predictions, presented here as experimental hypotheses, which we here simply state directly. Additional computational details, as required to test the hypotheses, are presented in the Data Analysis section below. As a convenience to the reader, Table 1 contains a glossary of the key symbols used in stating the hypotheses.

**H1** Consistent with the hypothesis of weak control along the GEM, one of the eigenvectors, ${\hat{e}}_{w}$, of the matrix B in Eq (11) will be nearly tangent to the GEM. That is, the weakly stable subspace, $\mathrm{span}\{{\hat{e}}_{w}\}$, will make an angle with the GEM of $\theta_{w}=\cos^{-1}\left({\hat{e}}_{t}\cdot {\hat{e}}_{w}\right)\approx 0$ (see Fig 3). Furthermore, the corresponding eigenvalue, *λ*_*w*_, will be well above 0, but less than 1 (i.e., 0 ≪ *λ*_*w*_ < 1).
**H2** In contrast, the fluctuation dynamics transverse to the GEM will be strongly stable: i.e., the eigenvalue *λ*_*s*_ satisfies 0 ≈ |*λ*_*s*_| ≪ *λ*_*w*_. The associated eigenvector, ${\hat{e}}_{s}$, and the strongly stable subspace $\mathrm{span}\{{\hat{e}}_{s}\}$, will be transverse (i.e., not tangent) to the GEM, but they need not be normal to it. That is, for $\theta_{s}=\cos^{-1}\left({\hat{e}}_{t}\cdot {\hat{e}}_{s}\right)$ we expect 0 ≈ *θ*_*w*_ ≪ *θ*_*s*_ (again, refer to Fig 3).
**H3** We expect the statistical persistence properties of the inter-trial fluctuations to be consistent with the stability properties of **H1** and **H2**. That is, the fluctuations in the weakly stable subspace will tend to persist over many trials, whereas those in the strongly stable direction will be corrected rapidly so that what remains is closely approximated by uncorrelated “white noise”. We characterize statistical persistence two ways: via the normalized lag-1 autocorrelation *R*(1), and via the exponent *α* from detrended fluctuation analysis (DFA). From Eq (20) and the subsequent discussion, we expect 0 ≈ |*R*_*s*_(1)| ≪ *R*_*w*_(1), whereas we expect the DFA exponents to satisfy 0.5 ≈ *α*_*s*_ ≪ *α*_*w*_.
**H4** For skilled performers we expect $\sigma_{e}/\sigma_{R}\approx s$ (Eq (9)), where the passive sensitivity *s* is the singular value of A at x* (Eq (8)), *σ*_*e*_ is the standard deviation of goal-level fluctuations (i.e., RMS error), and $\sigma_{R}$ is the standard deviation of goal-relevant fluctuations (Eq (7)). Combining this with local geometric stability analysis leads to the prediction that the goal-level error will scale with the intrinsic body-level noise according to Eq (19), repeated here for convenience:
  $$\frac{\sigma_{e}}{\sigma_{ns}}\approx \frac{\beta s}{\sqrt{1-\lambda_{s}^{2}}}\;≜\;s_{\text{TOT}},$$
  where *σ*_*ns*_ is the RMS value of the component of additive noise ***ν*** in the strongly-stable direction, *β* = sin(*θ*_*s*_) (Fig 3), and *s* is the passive sensitivity. For the shuffleboard task, *s* = *s*(*μ*), from Eq (8).

**Table 1:** Glossary of key symbols used in the statement of hypotheses H1–H4.

Symbol	Meaning	Where defined
${\hat{e}}_{t}$, ${\hat{e}}_{n}$	Unit vectors tangent and normal to the GEM	Eqs (5) and (6)
B	2 × 2 matrix of linearized state update equation	Eq (11)
*λ*_*w*_, *λ*_*s*_	Eigenvalues of B indicating weak and strong regulation of fluctuations near GEM	Eq (11) ff.
${\hat{e}}_{w}$, ${\hat{e}}_{s}$	Eigenvectors of B showing the weakly and strongly stable directions of inter-trial regulation	Eq (11) ff.
*θ*_*w*_, *θ*_*s*_	Angle between weak and strong eigenvectors of B and tangent to the GEM	Fig 3
*R*_*w*_(1), *R*_*s*_(1)	Lag-1 autocorrelations of weak and strong components of the body-level fluctuation time series	Eq (20)
*α*_*w*_, *α*_*s*_	DFA exponents of weak and strong components of body-level fluctuation time series	Eq (21) ff.
A	1 × 2 Jacobian of goal function evaluated at mean operating point (the body-goal variability matrix)	Eq (4)
*s*	Singular value of A (passive sensitivity to fluctuations near the GEM)	Eq (8)
*σ*_*e*_	Standard deviation of goal-level fluctuations (RMS task error)	Eq (9)
$\sigma_{R}$	Standard deviation of fluctuations normal to the GEM (RMS goal-relevant fluctuations)	Eq (9)
*σ*_*ns*_, *σ*_*nw*_	Standard deviations of the component of additive noise in the strongly and weakly stable directions.	Eq (17) ff.
*β*	sin(*θ*_*s*_)	Fig 3
$s_{\text{TOT}}$	Total body-goal sensitivity	Eq (19)

Hypotheses **H1**–**H3** can be tested directly by examining the eigenstructure of the matrix B in Eq (11). They are dynamical consequences of the more general hypothesis that Eq (11) is derived from a “GEM aware” controller, and hence strives to eliminate goal-relevant deviations quickly, after only one trial, while allowing goal-equivalent deviations to persist for multiple trials. In contrast, hypothesis **H4** emphasizes how the overall goal-level performance (as measured by *σ*_*e*_) will result from the interaction between the strongly-stable component of the intrinsic “input” noise (measured by *σ*_*ns*_), inter-trial error correction, and passive sensitivity.

The total body-goal sensitivity, *s*_TOT_, is an overall “gain” between body-level noise and goal-level error. We expect *λ*_*s*_ ≈ 0, and *β* = sin(*θ*_*s*_)<1 (Fig 3). Thus, $\beta /\sqrt{1-\lambda_{s}^{2}}$, which is the “active factor” of *s*_TOT_ will have a value on the order of unity. In contrast, the “passive factor” of *s*_TOT_, which is simply the passive sensitivity *s* (Eq (8)), may be substantially greater than unity. Thus, a somewhat counterintuitive effect of error-correcting control is that the passive sensitivity, which is determined by task properties *independent* from control, may play a dominant role in determining motor performance at the goal level.

### Experimental Apparatus and Protocol

**Fig 4:**
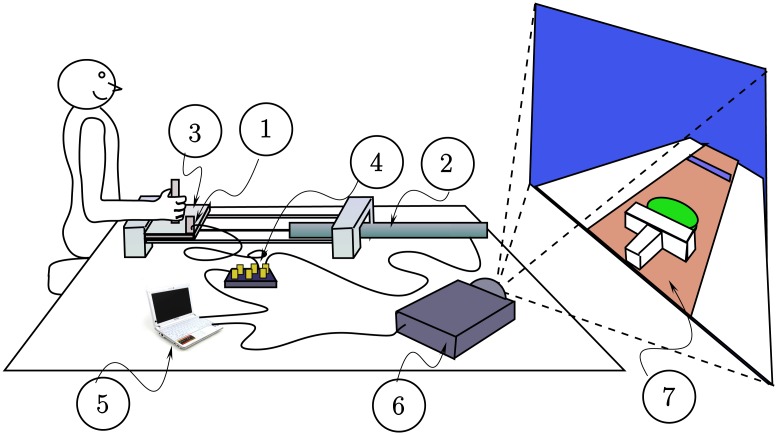
Schematic representation of the virtual shuffleboard game. The participant moves a manipulandum along a linear bearing. Position and acceleration data from the manipulandum is used to move a virtual shuffleboard cue that pushes a puck towards a target in the virtual world. The various parts of of setup are: (1) accelerometer; (2) LVDT (position sensor); (3) linear, low friction bearing; (4) data acquisition board; (5) control computer running LabVIEW (for data acquisition) and C++ modules (for graphics rendering and physics logic); (6) projector; (7) virtual environment projected on a screen.

Fig 4 shows a schematic representation of the experimental set-up for the shuffleboard game in a virtual environment. The participant was seated in an upright position, and in each trial moved a custom-built input device consisting of a manipulandum affixed to a low friction, single degree of freedom, linear bearing. Participants held the manipulandum with their dominant hand and pushed it in a direction parallel to the ground plane. The apparatus was configured for each participant so that at rest the upper arm was aligned with the midaxillary line and the angle between the upper arm and the forearm was approximately 90°.

Each trial started with the puck at *x* = 0 (recall Fig 1). The participant accelerated the manipulandum from rest. Position data was acquired from the manipulandum’s motion and used to generate the motion of a virtual shuffleboard cue in real time, via custom software, which pushed the puck on the virtual court. The release of the puck happened as the cue decelerated and the virtual contact force between the cue and the puck decreased to zero. At the point of release, the position and velocity, *x* and *v*, of the puck were acquired, defining the body state for a given trial. Thereafter, the acquired values of *x* and *v* were used to compute the motion of the puck as it slid on the virtual court and was decelerated by Coulomb friction before coming to rest. The movement of the shuffleboard cue and puck during the entire trial was generated in real time by the control software and projected onto a screen. Participants could see an animated 3D scene showing the movement of the puck on the court as it moved toward a visible target line before coming to a stop. The projector (InFocus LP70+) was located to the right and just behind the participants, approximately 3m from a 1.7m × 1.3m screen, with the settings adjusted for flicker-free images that filled the screen.

The position and velocity data were obtained from two transducers placed on the manipulandum and collected through two 12-bit channels: an accelerometer (ADXL320, Analog Devices, Inc., Norwood, MA) was used to collect acceleration data, which was integrated to provide the velocity; the other channel collected position data from a linear variable displacement transducer (LVDT) (Daytronic Corporation, Dayton, OH). The LVDT was also used to calibrate the accelerometer by scaling the doubly integrated acceleration signal to match the position signal. A National Instruments NIDAQCard-6024E data acquisition card was used to acquire the data to a laptop computer. A virtual instrument written in LabVIEW (National Instruments, Austin, TX) passed the velocity and position information in real time to a C++ program which used the Visualization Toolkit (VTK, http://www.vtk.org), an open-source graphics library, to render the 3D virtual environment. Both signals were sampled at 5kHz to provide smooth animation in the virtual environment. Even though the virtual environment has no physical units per se, we designed the system so that all VTK representations of lengths matched centimeters in the physical world: the accelerometer and LVDT were calibrated and data was recorded in cm/s^2^ and cm, respectively.

We expected the dynamical anisotropy predictions (**H1**–**H3**) to depend primarily on the local geometry of the GEM, and to not, therefore, depend on the friction coefficient *μ*. On the other hand, the scaling prediction, **H4**, depends on *μ* via the passive sensitivity, since *s* = *s*(*μ*) from Eq (8). Therefore, we had each participant perform the task with two different friction levels in the virtual world, giving a total of eight different participants/conditions. For a given velocity and position at release, the time of motion before the puck stops is inversely proportional to the coefficient of friction. We therefore selected values of *μ* so that the time for a hypothetical ideal trial varied uniformly between 3s and 5s. This ideal trial was defined by a release position of *x* = 0 and release velocity *v* determined from the goal function Eq (2) so that the puck would stop exactly at the target. The resulting set of 8 *μ* values were split into two sets: the lowest 4 gave “low friction” (LF) conditions, and the highest 4 “high friction” (HF) conditions. These different friction conditions gave us inter-trial data sets generated with different passive sensitivity properties, via Eq (8).

Four healthy, right-handed male participants aged 25, 28, 29 and 33 years (labeled P1–P4) participated in this study. Each participant was randomly assigned one HF and one LF friction condition to perform the shuffleboard task. The participants were instructed to launch the puck so that its center stopped on the target in every trial. Participants had the visual feedback from the 3D scene showing the error from a given trial. The goal-level error was also displayed momentarily on the screen providing a second, more precise, feedback on their performance. All participants were allowed to familiarize themselves with the task and the equipment, and practiced hitting the target until their average error *e* (Fig 1) over 50 trials was less than 10% of the target distance. That is, participants practiced until the average state $\bar{x}={\left(\bar{x},\bar{v}\right)}^{T}$ acquired over 50 trials lay within the error contours of Fig 2. All participants achieved this level of performance within four blocks of 50 trials.

**Fig 5:**
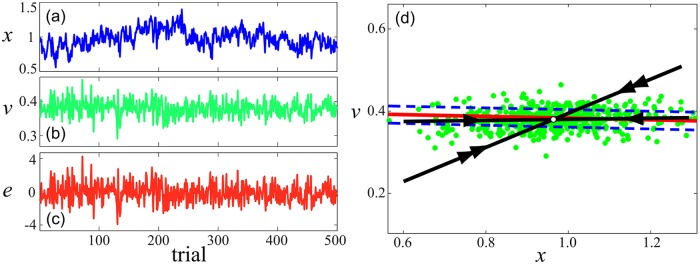
Typical data collected from one participant over 500 trials, for a given *μ* value. Plots (a–c): time series of position, velocity, and error at the target. The data is discrete, but plotted with lines to aid visualization. Plot (d): scatterplot of states **x** = (*x*, *v*)^T^ plotted as green dots. Also included for reference are the mean operating point x* (white dot), GEM (red curve), and ±10% goal-level error contours (dashed blue lines). The update matrix B (Eq (11)) is estimated from the inter-trial data via linear regression. The strongly (double arrow) and weakly (single arrow) stable subspaces obtained by solving the eigenvalue problem for B are shown as black lines. The weakly stable subspace is nearly parallel to the GEM tangent, while the strongly stable is at a much greater transverse angle (see Fig 3 for angle definitions).

Once the participants achieved the required level of performance, the data collection phase began. The body state **x** = (*x*, *v*)^T^ and goal-level error *e* were recorded for each trial. For each of the two friction conditions (LF and HF) the participant was required to perform 500 trials. All of the data was collected over three days: two days each of four 50-trial blocks, with two blocks before noon and two in the afternoon, followed by a day of two 50 trial blocks. Each block took no more than seven minutes and the participant was given up to five minutes of rest between blocks. The last block of P1-HF was incomplete due to an experiment malfunction, so only data from the first 9 blocks (450 trials) were subsequently analyzed; P3-HF had only 350 usable trials due to the entry of an erroneous friction coefficient. Typical inter-trial time series of states **x** = (*x*, *v*)^T^ obtained from one participant over 500 trials are shown in Fig 5(a)–5(c).

### Data Analysis

The complete data set for each of the 8 friction conditions (4 participants × 2 conditions each) consisted of time series of release position and velocity, ${\left\{x_{k}\right\}}_{k=1}^{N}$ and ${\left\{v_{k}\right\}}_{k=1}^{N}$, respectively, and the corresponding error, ${\left\{e_{k}\right\}}_{k=1}^{N}$, for each of *N* = 500 trials. The data was rescaled into dimensionless form, as for the goal function of Eq (2). Note, however, that the stability and persistence properties studied here depend only on the temporal relations between consecutive trials, so the rescaling does not affect the results presented in this paper. Except as noted, all data analyses were performed using Matlab (Mathworks, Natick, MA). All data and software used for this study is contained in Supporting Information S1 Data and Code.

The sample mean body state $\bar{x}={\left(\bar{x},\bar{v}\right)}^{T}$ over all trials was used to define the operating point used in Eq (4): that is, we took $x^{*}\equiv \bar{x}$. Fluctuation time series were then obtained from $u_{k}=x_{k}-\bar{x}$, and Eq (11) was used to estimate B via linear regression. That is, we used ordinary least squares to minimize the single-step mean-square prediction error 〈(**u**_*k*+1_ − B**u**_*k*_)^T^(**u**_*k*+1_ − B**u**_*k*_)〉, where, again, the angle brackets denote the ensemble average. A requirement for the use of this straightforward approach to estimation [60–62] is that the state measurement error or “noise” (as distinct from the process noise ***ν***_*k*_ in Eq (11)) not be too large. While there is no firm cutoff for how much measurement noise becomes problematic, Kantz and Screiber suggest (see [62], p. 251 ff.) that ordinary least squares works well as long as the measurement errors are under about 10%. In our case the measurement precision after calibration was approximately 2%, well under the suggested cutoff. Furthermore, we cross validate the estimate of B by comparing its eigenvalues against the lag-1 autocorrelation, which is computed independently, as discussed previously following Eq (21).

The eigenvectors of B, $\left\{{\hat{e}}_{w},{\hat{e}}_{s}\right\}$, and their corresponding eigenvalues, {*λ*_*w*_, *λ*_*s*_}, were then obtained as solutions to $B\hat{e}=\lambda \hat{e}$. A typical result of this eigenanalysis is shown in Fig 5(d). The alignment of the eigenvectors to the GEM was computed using the theoretical tangent vector from Eq (5) (recall the schematic of Fig 3). Because the empirically-determined operating point $\bar{x}$ was always close to, but never exactly on the GEM, as a check we also computed the eigenvector orientation using the tangent to the error contour passing through the operating point (determined from by $f\left(\bar{x}\right)=\bar{e}$, where *f* is the goal function Eq (2)). This was found to give identical results, confirming the closeness of $\bar{x}$ to the GEM. Together with the alignment information so obtained, the estimated eigenvalues of B, which quantify the stability of the inter-trial dynamics, were used to test **H1** and **H2**.

Next, the fluctuation time series ${\left\{u_{k}\right\}}_{k=1}^{N}$ in the original position-speed coordinates were transformed into time series ${\left\{z_{k}\right\}}_{k=1}^{N}$ expressed in eigencoordinates, via the linear coordinate transformation Eq (12). Following the discussion surrounding Eqs (20) and (21), statistical persistence in both directions was quantified using the lag-1 autorcorrelations *R*_*w*_(1) and *R*_*s*_(1), as well as the DFA exponents *α*_*w*_ and *α*_*s*_. These results allowed us to test **H3**.

To test the scaling relationship of **H4**, the RMS goal-level error *σ*_*e*_ was computed directly from the time series, ${\left\{e_{k}\right\}}_{k=1}^{N}$. Using Eq (8), the value of *μ* for a given set of trials, and the velocity component of the average operating point, $\bar{v}\equiv v^{*}$, we obtained an estimate of *s*. The values of *β* and *λ*_*s*_ were available from the eigenanalysis. For *σ*_*ns*_, we used the estimated B and Eq (12) to compute the residual of the regression expressed in eigencoordinates, via **r**_*k*_ = E^−1^(**u**_*k*+1_ − B**u**_*k*_). We then took $\langle |r_{s,k}^{\;2}|\rangle$ as an estimate of *σ*_*ns*_, where *r*_*s*,*k*_ is the strongly stable component of **r**_*k*_. Using these estimates to evaluate Eq (19) allowed us to test **H4**.

All of the above analyses depend critically on the eigenvalues and eigenvectors of the matrix B. To estimate B via regression we require only data from a set of trials, which need not themselves be consecutive, together with the subsequent states that are presumed to follow under the action of B via Eq (11). To eliminate the spurious “state update” between the last trial in each block and the first trial in the next block, we only consider the first 49 trials within each 50 trial block. In addition, to avoid possible transient “retraining” effects at the beginning of each block, we removed the first 4 trials, leaving 45 trials within each block, for a total of 450 trials per friction condition. Finally, to overcome known problems associated with the sensitivity of eigenvalue and eigenvector estimates to matrix errors [31], such as are unavoidable with matrices estimated via regression, we used bootstrapping [32–34] to estimate the various quantities needed to test our hypotheses.

For each iterate of the bootstrap, we selected a uniformly-distributed random sample of 450 states (with replacement) from the 450 available for each friction condition, together with the state from the next trial. In this way, we obtained an ensemble of “current states” (**x**_*k*_) and an ensemble of the corresponding “next states” (**x**_*k*+1_) that were used to obtain *one* estimate of B via linear regression. This estimate of B was then used to compute one set of eigenvalues and eigenvectors. The eigenvectors were then used to obtain the fluctuation components in the weakly and strongly stable directions, *z*_*w*_ and *z*_*s*_, via the transformation Eq (12). These allowed us to estimate the lag-1 autocorrelations using Eq (20). By choosing many such random samples, each resulting in its own estimate of B, we were able to generate an empirical probability distribution for all quantities needed to test **H1** and **H2**, and to partially test **H3** using *R*(1). The bootstrapping gave us reliable estimates of mean values together with 95% confidence intervals. For the above results, we used 10000 bootstrap iterates.

Since DFA relies on the proper temporal sequence of an entire data set (not just over a single lag as for the autocorrelation), the sampling procedure outlined above could not be used. In addition, because DFA does not give reliable estimates for small data sets, we concatenated all 10 trial blocks, again with the first four trials removed, and analysed the resulting data set of 460 trials at once. Such a concatenation procedure was shown in an analysis of Parkinsonian gait [63], using data sets of 25 strides each, to give results with sufficient accuracy to distinguish Parkinsonian and healthy participants. While perhaps not accurate enough to characterize subtle differences in long-range correlated data sets, as stated earlier this is emphatically *not* our aim here: we merely use DFA to provide a convenient, lag-independent measure of statistical persistence, which we checked against the lag-1 autocorrelation for consistency. For this paper, once the eigenvectors were found within each iterate of the bootstrap, the entire time series of fluctuations was transformed into eigencoordinates, again via Eq (12). The DFA exponents, *α*_*w*_ and *α*_*s*_, for the two eigencoordinate fluctuations were then obtained, allowing us to complete the test of **H3**. To reduce the computation time required to carry out 10000 DFA calculations for each friction condition, we used a version of the algorithm written in C [64], that was then called from Matlab.

Finally, to test **H4**, another variant of the bootstrap was used. In each bootstrap iteration, 450 samples with replacement were drawn and used to estimate *σ*_*e*_, *σ*_*ns*_, *s*, *β* and *λ*_*s*_, as needed for Eq (19); this was done for all 8 friction conditions. Within this bootstrap iteration, regression was then used to estimate the parameters *a* and *b* of a fit *σ*_*e*_/*σ*_*ns*_ = *as*_TOT_ + *b*: following Eq (19), we expected *a* ≈ 1 and *b* ≈ 0. Thus, after repeating this process 10000 times, we obtained estimates and confidence intervals for the slope *a* and y-intercept *b*, as required to test **H4**.

## Results

**Fig 6:**
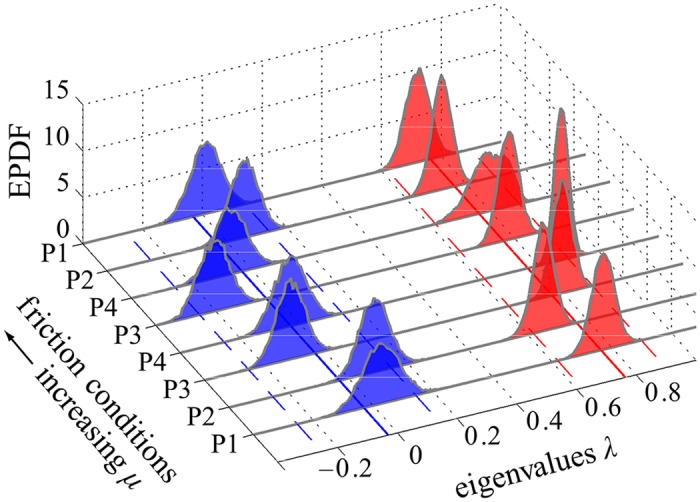
Empirical probability density functions (EPDFs) obtained via bootstrapping for eigenvalues *λ*_*w*_ (red) and *λ*_*s*_ (blue) of B (Eq (11)), each plotted vs. participant/condition. We see that 0 ≈ *λ*_*s*_ ≪ *λ*_*w*_ in all cases (aggregate mean *λ*_*s*_ = −0.03 with 95% CI of [−0.24, 0.14] and *λ*_*w*_ = 0.76 with 95% CI of [0.62, 0.90]), indicating much more vigorous inter-trial control in the strong direction than in the weak. Bootstrapping was carried out using 10000 random samples of 450 trials each, with replacement, from the complete data set, with the final and first four trials removed from each 50 trial block. The solid lines in the horizontal plane shows the aggregate mean value, and the dashed lines indicate the aggregate 95% CI, as reported above.

**Fig 7:**
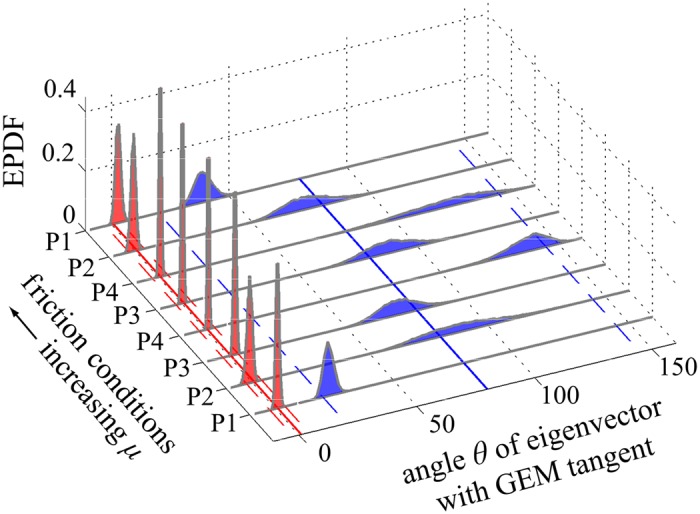
EPDFs for the angles *θ*_*w*_ (red) and *θ*_*s*_ (blue) between the eigenvectors ${\hat{e}}_{w}$ and ${\hat{e}}_{s}$ of B, respectively, and the unit tangent ${\hat{e}}_{t}$ (Eq (5)), each plotted vs. participant/condition. All other figure details are as in Fig 6. We see that in all cases 0 ≈ |*θ*_*w*_| ≪ *θ*_*s*_ (*θ*_*w*_ = 0.90° [−2.36°, 3.99°] and *θ*_*s*_ = 79.75° [20.66°, 144.75°]). The orientation of the weakly stable subspace was found to be nearly tangent to the GEM, with a small range of variation, whereas the strongly stable subspace made a much greater angle with the GEM and varied substantially. Together with the results of Fig 6, these results confirm hypotheses **H1** and **H2**.

Fig 6 shows empirical probability density functions (EPDFs), obtained using bootstrapping, for the eigenvalues {*λ*_*w*_, *λ*_*s*_} of the matrix B (Eq (11)). We see that in all cases they satisfy 0 ≈ |*λ*_*s*_| ≪ *λ*_*w*_ < 1. In aggregate, across all participants (P1–P4) and friction conditions, we found *λ*_*s*_ = −0.03 [−0.24, 0.14] and *λ*_*w*_ = 0.76 [0.62, 0.90], where here and throughout the stated estimate is the aggregate mean, and the closed interval represents the aggregate 95% confidence interval (CI). The orientation of the eigenvectors is shown in Fig 7, which plots the EPDFs for the angles $\theta_{w}=\cos^{-1}\left({\hat{e}}_{w}\cdot {\hat{e}}_{t}\right)$, and $\theta_{s}=\cos^{-1}\left({\hat{e}}_{s}\cdot {\hat{e}}_{t}\right)$. We see that, for all participants/conditions, the weakly stable eigenvector was very close to the tangent, and the strongly stable eigenvector made a larger transverse angle with it, so that 0 ≈ |*θ*_*w*_| ≪ *θ*_*s*_. Specifically, we found *θ*_*w*_ = 0.90° [−2.36°, 3.99°] and *θ*_*s*_ = 79.75° [20.66°, 144.75°]. We note that the orientation of the weakly stable subspace is tightly regulated to be near the GEM’s tangent (i.e., its CI is small, spanning less than 7°), whereas the orientation of the strongly stable subspace is not tightly regulated (its CI spans over 124°). The aggregate values of the matrix components of B were found as B(1, 1) = 0.76 [0.62, 0.90], B(1, 2) = −0.26 [−2.03, 1.19], B(2, 1) = −0.01 [−0.04, 0.03], and B(2, 2) = −0.03 [−0.25, 0.14]. Using the mean matrix components as a simple consistency check, we found values of *λ*_*w*_ and *λ*_*s*_ equal to the means obtained via bootstrapping, above.

The results shown in Figs 6 and 7 strongly support hypotheses **H1** and **H2**. We found that the component of the inter-trial dynamics directed along the strongly stable subspace acted to quickly correct deviations off of the GEM that caused goal-level errors. For example, for the estimated mean value *λ*_*s*_ = −0.03, Eq (15) shows that a deviation transverse to the GEM would be, in the absence of noise, reduced to 3% of its initial magnitude after only one trial. In contrast, the dynamics in the weakly stable subspace did not rapidly correct deviations that were approximately tangent the GEM, and which therefore had little effect on error at the target. For the mean value of *λ*_*w*_ = 0.76, Eq (14) shows that, in the absence of noise, 9 iterates would be required to reduce an initial deviation to less than 10% of its initial value.

**Fig 8:**
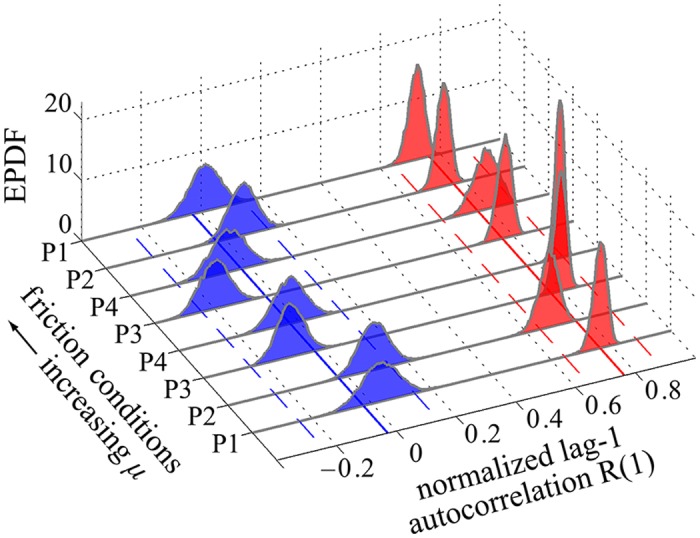
EPDFs for the normalized lag-1 autocorrelations *R*_*w*_(1) (red) and *R*_*s*_(1) (blue) for fluctuations in the weakly and strongly stable subspaces (Fig 3), respectively, plotted vs. participants/conditions. All other figure details are as in Fig 6. We find in all cases that 0 ≈ |*R*_*s*_(1)| ≪ *R*_*w*_(1) (*R*_*s*_(1) = −0.03 [−0.24, 0.14] and *R*_*w*_(1) = 0.76 [0.64, 0.88]). The results show strong positive correlation between successive fluctuations in the weakly stable direction, which is nearly tangent to the GEM (Fig 7), indicating that fluctuations persisted over multiple trials. In contrast, the strongly stable fluctuations were close to being uncorrelated, consistent with the action of strong control transverse to the GEM.

**Fig 9:**
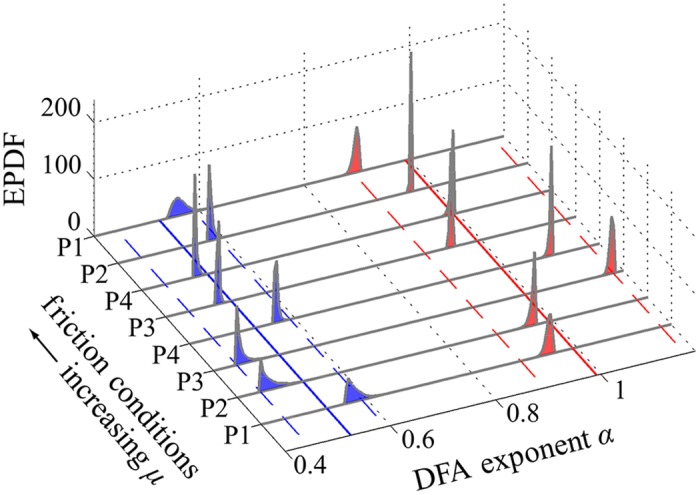
EPDFs for the DFA exponents *α*_*w*_ (red) and *α*_*s*_ (blue) for fluctuations in the weakly and strongly stable subspaces (Fig 3), respectively, plotted vs. participants/conditions. These calculations were carried out on the entire data set of fluctuations expressed in eigencoordinates, obtained via Eq (12) within each of 10000 bootstrap iterations. We found in all cases that 0.5 ≈ *α*_*s*_ ≪ *α*_*w*_ (*α*_*s*_ = 0.52 [0.44, 0.59] and *α*_*w*_ = 0.99 [0.89, 1.16]). The results indicate substantial persistence between successive fluctuations in the weakly stable direction, which is nearly tangent to the GEM (Fig 7), and nearly uncorrelated fluctuations in the strongly stable direction. These results, together with those of Fig 8, strongly confirm **H3**.

In Fig 8 we show the EPDFs obtained for the normalized lag-1 autocorrelations of fluctuations in the two eigendirections, for all friction participants/conditions. We find in all cases that 0 ≈ |*R*_*s*_(1)| ≪ *R*_*w*_(1). Specifically, we estimate *R*_*s*_(1) = −0.03 [−0.24, 0.14] and *R*_*w*_(1) = 0.76 [0.64, 0.88]. These results indicate that the trial-to-trial fluctuations in the weakly stable direction show greater persistence than those in the strongly stable direction. Furthermore, the strong control results in fluctuations that are close to uncorrelated white noise (since *R*_*s*_(1) ≈ 0). As anticipated in the discussion following Eq (21), these results are nearly identical to the local stability results in Fig 6. The EPDFs obtained for the DFA exponents *α*_*w*_ and *α*_*s*_ for fluctuations in the weakly and strongly stable subspaces, respectively, are shown in Fig 9. We found *α*_*s*_ = 0.52 [0.44, 0.59] and *α*_*w*_ = 0.99 [0.89, 1.16]. Thus, in all cases 0.5 ≈ *α*_*s*_ ≪ *α*_*w*_, showing substantial persistence between successive fluctuations in the weakly stable direction, and nearly uncorrelated fluctuations in the strongly stable direction. Thus, the persistence results of Figs 8 and 9 are consistent with each other and, taken together, strongly confirm **H3**.

**Fig 10:**
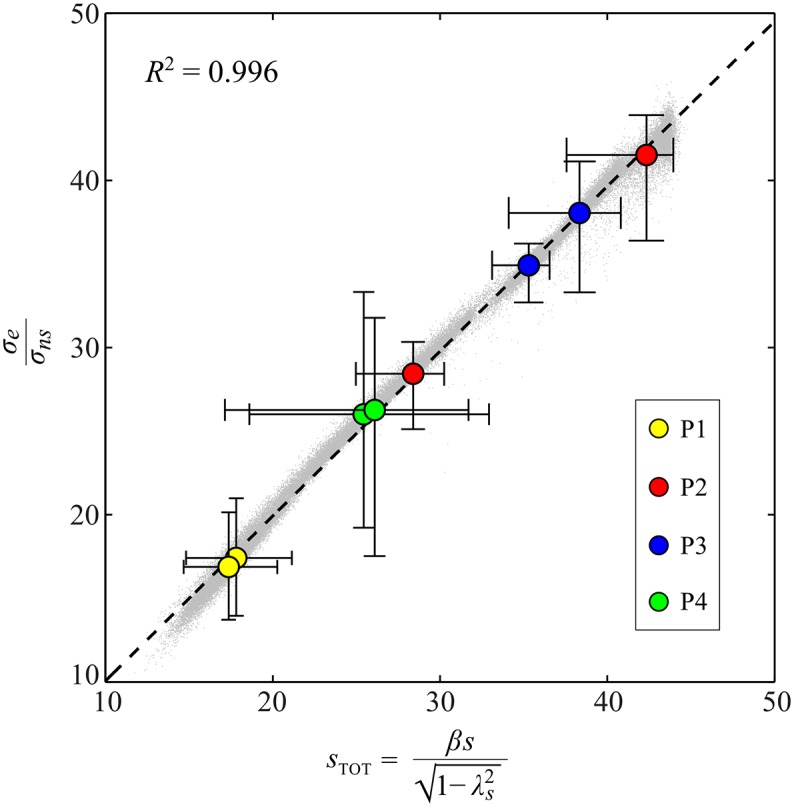
Plot of the variability ratio *σ*_*e*_/*σ*_*ns*_ vs. total body-goal sensitivity *s*_TOT_ (see Eq (19)). The light gray dots represent all values obtained by bootstrapping. One such point was generated for all 8 friction conditions within each of 10000 bootstrap iterations, and then linear regression gave estimates of the slope *a* and y-intercept *b*, yielding EPDFs for both. We found *a* = 0.99 [0.93, 1.03] and *b* = 0.21 [−0.98, 1.52], showing that *σ*_*e*_/*σ*_*ns*_ ≈ *s*_TOT_, which confirms hypothesis **H4**. The dashed line is plotted using the bootstrap mean values of *a* and *b*; *R*^2^ = 0.996 for the fit. Also shown for reference are the average values for each participant/condition individually, obtained via bootstrapping, with error bars indicating 95% CIs. These average values fall very close to the fit line.

Finally, Fig 10 illustrates how the variability ratio *σ*_*e*_/*σ*_*ns*_, which represents an empirical “gain” between intrinsic body-level noise and goal-level variability, was found to linearly scale with the total body-goal sensitivity *s*_TOT_ (Eq (19)). The light gray dots in the plot represent values obtained by bootstrapping: one such point was generated for all 8 friction conditions and linear regression was applied within each of 10000 iterations. This process yielded estimates for the slope, *a* = 0.99 [0.93, 1.03], and y-intercept, *b* = 0.21 [−0.98, 1.52]. The resulting aggregate fit had an *R*^2^ of 0.996. As a check, we used all 8 × 10000 points at once for a single linear fit; this did not change the fit parameters or the *R*^2^ value. The figure also includes the average values obtained for each participant/condition, computed independently by bootstrapping, together with error bars representing 95% CIs. The uneven size of the error bars, especially in the horizontal direction, reflects the nonlinearity of *s*_TOT_, particularly the factor of *β* = sin(*θ*_*s*_). We see that in each case the mean points fall very near the linear fit, indicating that the scaling relationship held not only in aggregate, but for each participant/condition individually. Indeed, similar fits done for each participant independently yielded *R*^2^ estimates of 0.962, 0.991, 0.979 and 0.992, values not meaningfully different from the overall value. Thus, we concluded that for all participants/conditions Eq (19) holds, confirming hypothesis **H4**.

We conclude this section with an illustration of how our approach overcomes the potential interpretive ambiguity stemming from the coordinate dependence of variance [28, 40]. As discussed when presenting Eqs (22) and (23), the dynamical analysis carried out here yields quantities that are intrinsic to the observed temporal fluctuations, and hence are coordinate invariant. As a demonstration of this invariance, and its advantage in analyzing motor variability, we constructed a “worst case” coordinate transformation similar in form to Eq (12). However, in this case we defined new fluctuation coordinates **q** = (*q*_1_, *q*_2_)^T^ via **u** = P**q**, where the matrix P was obtained from principal component analysis [42], as follows: let P = SC, in which C is a matrix with columns composed of the eigenvectors (i.e., the principal components) of the fluctuation covariance 〈**uu**^T^〉, and S is a diagonal matrix with the square root of the inverse principal values, 1/*σ*_1_ and 1/*σ*_2_, along its diagonal. The result of applying this transformation is that both of the new coordinates *q*_1_ and *q*_2_ have identical variance, and hence the variance “cloud” in the (*q*_1_, *q*_2_) plane is isotropic by construction (i.e., the variance ellipse is a circle).

**Fig 11:**
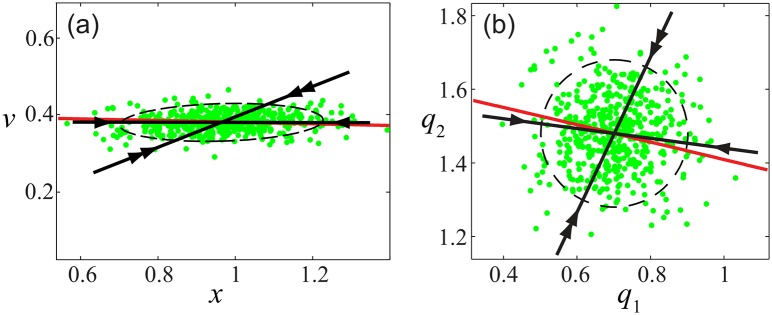
Illustration of the coordinate invariance of fluctuation dynamics near the GEM: (a) results for data in original (*x*, *v*) coordinates, showing an anisotropic variance ellipse (dashed line) with principal axes equal to the square root of the principal values; (b) results for data transformed using rescaled principal coordinates (*q*_1_, *q*_2_), showing an isotropic variance ellipse (i.e., a circle). Both figures contain the same data (green dots), GEM (red line), and strongly stable (double arrow) and weakly stable (single arrow) subspaces (black lines). We see that the local stability analysis consistently represents the organization of control around the GEM, whereas the ratio of variances normal and tangent to the GEM clearly fails to identify the GEM location in plot (b).

Fig 11 shows what happens when we apply this transformation to typical data from a single participant and friction condition. In Fig 11(a) we see the original data and the local stability results estimated from it, whereas in Fig 11(b) we see the equivalent analysis carried out on the transformed data. The eigenvalues obtained are identical in both cases, since the original matrix, B (Eq (11)), and the transformed matrix, P^−1^BP, are congruent. Furthermore, as discussed following Eq (23), the transformed eigenvectors maintain their qualitative relationship with the transformed GEM: that is, the weakly stable subspace is nearly tangent to the GEM, whereas the strongly stable subspace is transverse to the GEM at a much greater angle. Thus, in both cases 0 ≈ *θ*_*w*_ ≪ *θ*_*s*_ so that the local stability picture is qualitatively unchanged by the coordinate transformation, and can be used to test a candidate GEM in either case. In sharp contrast, using the shape of the variance ellipse to identify the GEM location works reasonably well for Fig 11(a), but clearly fails for the case shown in Fig 11(b). Indeed, using an approach similar to that used to create Fig 11(b), one can change the shape of the variance ellipse at will, while in all cases maintaining the proper qualitative relationship between the GEM and the weakly and strongly stable subspaces.

## Discussion

Understanding how humans are able to perform accurate and repeatable goal-directed movements in the presence of inherent biological noise [7–11] and neuromotor redundancy [22–24] has been a critical goal of neuroscience research (e.g., [45, 46, 48]) since the pioneering work of Bernstein [1]. In recent years, studies addressing this question have focused on using either task manifold ideas to address redundancy (e.g., [12–14]), or time series analysis methods to study temporal correlation structure (e.g., [25, 51, 54, 55]).

However, these often divergent perspectives have not yet been fully unified into a comprehensive theoretical framework, and it remains an open question whether these various aspects of inter-trial variability represent distinct neurophysiological phenomena, or can be traced back to a single underlying motor regulation process. The work in this paper expands on previous efforts [25, 28] suggesting that such a unification can be achieved by considering the inter-trial dynamics of fluctuations near a task’s goal equivalent manifold (GEM). These studies have shown that a fundamental feature of such inter-trial fluctuations is that they are dynamically anisotropic in a manner that respects the local geometry of the GEM [25–29], an observation supported by work carried out from different task manifold perspectives [30, 54, 65].

Using a custom-built interactive virtual environment, we studied the variability exhibited by skilled participants as they carried out repeated trials of a simple shuffleboard game. The experiments were used to test theoretical predictions obtained from a new analysis, presented in this paper, of a previously-developed general model for inter-trial error correction [25, 28]. The assumption of skilled performance, for which body states will remain close to the GEM, yields a simple linear inter-trial control model. The further empirically-supported assumption that inter-trial error correction satisfies a generalized interpretation of the minimum intervention principle (MIP), together with an analysis of geometric stability, yielded theoretical predictions about the geometrical and temporal structure of inter-trial variability, showing analytically how body-level variability generates variability at the goal level. In particular, we showed that the assumptions underlying our analysis give rise to a new scaling relationship (Eq (19)), which introduces the total body-goal sensitivity, *s*_TOT_, a quantity showing how intrinsic goal-relevant fluctuations at the body level are mapped into fluctuations at the goal level. This relationship provides a unification of task manifold, control theoretic, and dynamical (time series) perspectives by showing specifically how the GEM geometry, passive sensitivity, and active error correction combine to yield task performance.

The predictions resulting from our analysis were summarized in the form of four experimental hypothesis, which were tested using data from four participants playing the shuffleboard game. To demonstrate the generality of the dynamical anisotropy predictions (**H1**–**H3**), and, more importantly, to allow us to tease apart active and passive effects in task performance as specified by the scaling prediction **H4**, we had each participant perform the task with two different friction levels, giving a total of eight different participants/conditions. All of our hypotheses were very strongly confirmed: in all cases, the difference between local stability and correlation properties in the weakly and strongly stable directions was just as predicted by theory (Figs 6–9), confirming **H1**–**H3**; and the goal-level performance scaled as predicted across all participants and friction conditions (Fig 10), confirming **H4**.

Given the nature of **H4**, which concerns the scaling relationship Eq (19) and therefore depends on all assumptions used in its derivation, these experimental results do more than characterize the behavior for these particular participants executing this particular task. Rather, they serve to validate our general model for inter-trial error-correcting control near the GEM. Thus, while this work does not make any direct ties to underlying physiological mechanisms, our results indicate that the combined geometrical and temporal structure of observed fluctuations can be explained by a single, relatively simple process. This supports the idea that one need not posit separate neurophysiological mechanisms for controlling such disparate features as the geometric distribution of trials about the GEM, the stability of inter-trial fluctuations, and the goal-level performance, but, rather, that all such behaviors arise from a single, unified process of error regulation in the presence of task-level redundancy.

Another contribution of this paper is the introduction of statistical bootstrapping [32–34] to the analysis of movement variability data. Using this approach, we were able to estimate the underlying probability distribution for quantities required by each hypothesis (e.g., eigenvalues, correlations, etc.), thus demonstrating that the predicted dynamical anisotropy is very highly significant in each case individually (Figs 6–9), without the need for conventional significance testing. Furthermore, this data analysis allowed us to confirm the theoretical performance scaling prediction (Fig 10) to high precision, thus demonstrating that task performance was largely determined by passive sensitivity, which in this case was a function of the friction condition (Eq (8)). This theoretical prediction is perhaps counterintuitive, because the passive sensitivity is determined entirely by the task’s goal function (Eq (2)), *independent* from any consideration of control. However, this behavior occurs precisely because error-correcting control strongly compresses variability onto the GEM. Thus, as shown theoretically by using Eq (18) in Eq (16) (with the understanding that *λ*_*s*_ ≈ 0, as shown in Fig 6), the scale of goal-relevant fluctuations is minimized, taking a value proportional to the scale of the strongly-stable component of the intrinsic noise. Therefore, for skilled participants, the resulting performance (as measured by the RMS error at the goal) is largely determined by the passive sensitivity, which is a property of the task as defined by the goal function.

Finally, as shown in our theoretical discussion and demonstrated with our experimental data, the dynamical approach used for this study yields results that are invariant under quite general (differentiable and invertible) coordinate transformations, something that is not true for variability analyses based only on the spatial distribution of body states near a given task manifold. Even in the “worst case” scenario for which coordinates are chosen that render the variability cloud isotropic, so that it contains *no* information about the location of the GEM, as shown in Fig 11, the dynamical approach yields correct information about the structure of inter-trial fluctuations. Thus, our data analysis methods resolve the persistent problem of coordinate dependence of variability measures [30, 40]. This suggests that the dynamical coordinates, as obtained via the transformation Eq (12), provide a set of objective, canonical coordinates for the study of inter-trial variability: that is, they represent coordinates that are intrinsic to the regulatory process responsible for inter-trial error correction.

These findings again highlight the critical importance of considering fluctuation dynamics [25–27, 30, 51–54] in both theoretical and experimental studies aimed at understanding the neuromuscular control of complex movements. While time series analyses alone can yield important descriptive information, in the absence of any underlying model they often have limited explanatory power. Recent efforts have seen the use of time series analyses to interpret model outputs and/or predictions [46, 48, 54, 66]. These efforts have yielded findings qualitatively similar to ours, and consistent with our interpretations of inter-trial variabilty presented both here and elsewhere [25, 26, 28, 29]. Even though these efforts have focused on motor learning, which we do not, conceptually there is a strong affinity between these papers and the work presented here. In [46, 54, 66], van Beers and colleagues used simple linear models with direct error feedback to analyze task performance when reaching to a point [46, 66] or a line [54]. Their lag-1 autocorrelation analyses for the redundant task of reaching to a line showed strong statistical persistence along the target line and uncorrelated fluctuations perpendicular to it, precisely as we would theoretically predict and very similar to our own findings (our Figs 8 and 9). In parallel work, Abe & Sternad [30] also obtained similar results applying both lag-1 autocorrelation and DFA analyses to van Beers’ model of the same task. Both studies thus independently support the experimental results presented here.

The analytical formalisms presented in the present paper, however, add several important extensions to these experimental observations. First, here we tie these time series analysis approaches directly to the stability properties of the dynamical system that generates the observed fluctuations, as determined by its eigenvalues and eigenvectors (Figs 6 and 7). Second, by formally defining the task in terms of a goal function (Eq (2)), we are able to show analytically (Eq (19)) how active and passive properties of the task interact to affect goal level fluctuations, a theoretical prediction that we test and confirm experimentally (Fig 10). Finally, van Beers’ model accounts only for the correction of goal-relevant errors, that is, of body-level fluctuations perpendicular to the GEM, and thus implements an ideal MIP-based controller with no control acting along the task manifold. However, as we have shown in previous work using models derived using a stochastic optimal control framework [25], and as discussed here and demonstrated experimentally by us [28] and others [36], such “pure” MIP controllers are not observed experimentally: that is, we find that the fluctuations along the GEM do not exhibit an unbounded random walk. Furthermore, our approach allows us to demonstrate this deviation from ideal MIP behavior geometrically, as well as in terms of stability and correlation properties. A conclusion of our work is that, while the control observed experimentally is congruent with the task manifold, it is not perfectly aligned with it: instead, the direction of “minimum intervention” (i.e., of weakest control) is close to, but not exactly tangent to the GEM. Nor is the direction of strongest control necessarily perpendicular to the GEM. One possible interpretation of these observations is that there are other competing costs, beyond simple error correction, that are at play during repeated task execution.

Other recent attempts to connect temporal analyses to task manifold geometry [27, 51] have similarly supported our experimental findings, but have not directly shown how such results can be predicted from a general model-based analysis, as the current work does. Dingwell et al. [27] applied lag-1 correlation analyses to a redundant reaching task, but did not directly connect those experimental analyses back to any underlying computational model. Rácz & Valero-Cuevas [51] used DFA analyses on data from a redundant, 3-finger grasping task to provide an experimental demonstration of the need to consider control as acting across both spatial and temporal domains. However, their work again did not provide mathematical theory able to explain and predict the observed behaviors. Nevertheless, in spite of these differences in experimental and/or computational approaches, each of the studies described above obtained findings consistent with our conclusion that the diverse geometrical and temporal aspects of inter-trial variability likely derive from a single underlying motor regulation process.

Our approach fully integrates task manifold geometry with ideas from control and dynamical systems theory, and thereby can be used to explain the structure of observed motor variability from a model-based, theoretical perspective. The theory and methods presented in this paper are quite general, and should be applicable to the study of skilled motor performance for a wide range of discrete, or discretizable, tasks. That said, general application can be expected to encounter difficulties, especially for tasks in which the relevant body and/or goal variables are high-dimensional (so that visualizing the GEM is difficult, if not impossible), as well as for tasks in which the goal function and GEM are not readily available in analytical form. In such cases, the basic theory will have to be used to formulate suitable, purely abstract, computational methods.

The assumption of skilled motor behavior, which implies that all fluctuations are near the GEM, permitted us to employ linear mathematics in our study. Without this linearity, it would have been much more difficult to make such precise, analytically-derived predictions. However, we did not impose linearity as a mere analytical convenience. On the contrary, our results show that a linear model of “GEM-aware” error correction captures key facets of the observed variability structure with substantial accuracy. The main aims of this paper were to robustly demonstrate the nature of dynamic anisotropy, to show how task performance is generated by the interaction of the GEM geometry and inter-trial error correction, and to demonstrate that such an approach yields results that are not sensitive to the coordinates chosen. As such, our focus on the steady state (i.e., learned) behavior of the inter-trial regulation system was appropriate. But this does not mean that the models and methods presented here would not have value for studies related to motor learning. Indeed, as discussed at some length above, models with a very similar mathematical structure have been used to precisely that end. From a dynamical systems perspective, our approach treats skilled movements as a “stochastic attractor” of the more general perception-action system engaged in motor learning. A logical point of departure for future work aimed at extending our methods to motor learning would be to study how the the “transient” portion of the a learning data set approaches the “steady-state” local geometrical structure uncovered using the methods of this paper. While such explorations would no doubt pose multiple challenges, in principle the theoretical concepts presented here could be extended to address questions of learning and/or adaptation, topics that we see as interesting aims of future work.

## Supporting Information

S1 Data and Code A compressed folder containing all data and software used for this study. (ZIP)

## References

[1] Bernstein NA. The Coordination and Regulation of Movement. London: Pergamon Press; 1967.

[2] Newell KM, Corcos DM, editors. Variability and Motor Control. Champaign, IL: Human Kinetics; 1993.

[3] Davids K, Benett S, Newell K, editors. Movement System Variability. Champaign, IL: Human Kinetics; 2006.

[4] Latash M. There is no motor redundancy in human movements. There is motor abundance. Motor Control. 2000;4:259–260. 10.1123/mcj.4.3.259 10970151

[5] van Emmerik REA, van Wegen EEH. On the Functional Aspects of Variability in Postural Control. Exercise and Sport Sciences Reviews. 2002;30(4):177 12398115 10.1097/00003677-200210000-00007

[6] Davids K, Glazier P, Araújo D, Bartlett R. Movement Systems as Dynamical Systems: The functional role of variability and its implications for Sports Medicine. Sports Medicine. 2003;33(4):245–260. 10.2165/00007256-200333040-00001 12688825

[7] Stein RB, Gossen ER, Jones KE. Neuronal Variability: Noise or Part of the Signal? Nature Reviews Neuroscience. 2005;6:389–397. 10.1038/nrn1668 15861181

[8] Osborne LC, Lisberger SG, Bialek W. A Sensory Source for Motor Variation. Nature. 2005;437:412–416. 10.1038/nature03961 16163357 PMC2551316

[9] Faisal AA, Selen LPJ, Wolpert DM. Noise in the nervous system. Nature Reviews Neuroscience. 2008;9(4):292–303. 10.1038/nrn2258 18319728 PMC2631351

[10] Eldar A, Elowitz MB. Functional roles for noise in genetic circuits. Nature. 2010;467(7312):167–173. 10.1038/nature09326 20829787 PMC4100692

[11] McDonnell MD, Ward LM. The benefits of noise in neural systems: bridging theory and experiment. Nature Reviews Neuroscience. 2011;12(7):415–426. 10.1038/nrn3061 21685932

[12] Scholz JP, Schöner G. The Uncontrolled Manifold Concept: Identifying Control Variables for a Functional Task. Experimental Brain Research. 1999;126(3):289–305. 10.1007/s002210050738 10382616

[13] Müller H, Sternad D. Decomposition of Variability in the Execution of Goal-Oriented Tasks: Three Components of Skill Improvement. Journal of Experimental Psychology: Human Perception and Performance. 2004;30(1):212–233. 14769078 10.1037/0096-1523.30.1.212

[14] Cusumano JP, Cesari P. Body-goal variability mapping in an Aiming Task. Journal of Biological Cybernetics. 2006;94(5):367–379. 10.1007/s00422-006-0052-1 16501988

[15] Scholz J, Schöner G, Latash M. Identifying the control structure of multijoint coordination during pistol shooting. Experimental Brain Research. 2000;135:382–404. 10.1007/s002210000540 11146817

[16] Latash ML, Scholz JP, Schöner G. Motor Control Strategies Revealed in the Structure of Motor Variability. Exercise & Sport Sciences Reviews. 2002;30(1):26–31. 11800496 10.1097/00003677-200201000-00006

[17] Schöner G, Scholz JP. Analyzing Variance in Multi-Degree-of-Freedom Movements: Uncovering Structure Versus Extracting Correlations. Motor Control. 2007;11(3):259–275. 10.1123/mcj.11.3.259 17715459

[18] Cohen RG, Sternad D. Variability in motor learning: relocating, channeling and reducing noise. Experimental Brain Research. 2009;193(1):69–83. 10.1007/s00221-008-1596-1 18953531 PMC2756422

[19] Ranganathan R, Newell KM. Influence of Motor Learning on Utilizing Path Redundancy. Neuroscience Letters. 2010;469(3):416–420. 10.1016/j.neulet.2009.12.041 20035835

[20] Sternad D, Abe MO, Hu X, Müller H. Neuromotor noise, error tolerance and velocity-dependent costs in skilled performance. PLoS Computational Biology. 2011;7(9):e1002159 10.1371/journal.pcbi.1002159 21966262 PMC3178634

[21] John J, Cusumano JP. Inter-Trial Dynamics of Repeated Skilled Movements. In: Proceedings of the ASME International Design Engineering Technical Conference & Information in Engineering Conference, Vol. 1 Pts. A–C; 2008. p. 707–716.

[22] Scott SH. Optimal Feedback Control and the Neural Basis of Volitional Motor Control. Nature Reviews Neuroscience. 2004;5(7):532–546. 10.1038/nrn1427 15208695

[23] Todorov E, Jordan MI. Optimal feedback control as a theory of motor coordination. Nature Neuroscience. 2002;5(11):1226–1235. 10.1038/nn963 12404008

[24] Todorov E. Optimality principles in sensorimotor control. Nature Neuroscience. 2004;7(9):907–915. 10.1038/nn1309 15332089 PMC1488877

[25] Dingwell JB, Cusumano JP. Re-interpreting detrended fluctuation analyses of stride-to-stride variability in human walking. Gait & Posture. 2010;32(3):348–353. 10.1016/j.gaitpost.2010.06.004 20605097 PMC2942973

[26] Dingwell JB, John J, Cusumano JP. Do Humans Optimally Exploit Redundancy to Control Step Variability in Walking? PLoS Computational Biology. 2010;6(7):185–205. 10.1371/journal.pcbi.1000856 PMC2904769 20657664

[27] Dingwell JB, Smallwood RF, Cusumano JP. Trial-to-trial dynamics and learning in a generalized, redundant reaching task. Journal of Neurophysiology. 2013;109(1):225–237. 10.1152/jn.00951.2011 23054607 PMC3545167

[28] Cusumano JP, Dingwell JB. Movement variability near goal equivalent manifolds: Fluctuations, control, and model-based analysis. Human Movement Science. 2013;32(5):899–923. 10.1016/j.humov.2013.07.019 24210574 PMC3858478

[29] Cusumano JP, Mahoney JM, Dingwell JB. The Dynamical Analysis of Inter-Trial Fluctuations Near Goal Equivalent Manifolds. Advances in Experimental Medicine and Biology. 2014;826:125–145. 10.1007/978-1-4939-1338-1_9 25330889 PMC9128735

[30] Abe MO, Sternad D. Directionality in Distribution and Temporal Structure of Variability in Skill Acquisition. Frontiers in Human Neuroscience. 2013;7(225). 10.3389/fnhum.2013.00225 23761742 PMC3674323

[31] Golub GH, Van Loan CF. Matrix Computations. Baltimore, MD: The John Hopkins University Press; 1996.

[32] Akman OE, Broomhead D, Clement R, Abadi R. Nonlinear time series analysis of jerk congenital nystagmus. Journal of Computational Neuroscience. 2006;21(2):153–170. 10.1007/s10827-006-7816-4 16732490

[33] Press WH, Teukolsky SA, Vetterling WT, Flannery BP. Numerical Recipes in C: The Art of Scientific Computing. Cambridge, UK: Cambridge University Press; 1992.

[34] Efron B, Tibshirani RJ. An Introduction to the Bootstrap vol. 57 of CRC Monographs on Statistics & Applied Probability. Boca Raton, FL: Chapman & Hall; 1994.

[35] Domkin D, Laczko J, Jaric S, Johansson H, Latsh M. Structure of joint variability in bimanual pointing task. Experimental Brain Research. 2002;143:11–23. 10.1007/s00221-001-0944-1 11907686

[36] Valero-Cuevas FJ, Venkadesan M, Todorov E. Structured variability of muscle activations supports the minimal intervention principle of motor control. Journal of neurophysiology. 2009;102(1):59 10.1152/jn.90324.2008 19369362 PMC2712269

[37] Krishnamoorthy V, Latash ML, Scholz JP, Zatsiorsky VM. Muscle synergies during shifts of the center of pressure by standing persons. Experimental brain research. 2003;152(3):281–292. 10.1007/s00221-003-1574-6 12904934

[38] Latash ML, Scholz JF, Danion F, Schoner G. Structure of motor variability in marginally redundant multifinger force production tasks. Experimental Brain Research. 2001;141(2):153–165. 10.1007/s002210100861 11713627

[39] Kang N, Shinohara M, Zatsiorsky VM, Latash ML. Learning multi-finger synergies: an uncontrolled manifold analysis. Experimental Brain Research. 2004;157(3):336–350. 10.1007/s00221-004-1850-0 15042264

[40] Sternad D, Park SW, Müller H, Hogan N. Coordinate Dependence of Variability Analysis. PLoS Computational Biology. 2010;6(4):e1000751 10.1371/journal.pcbi.1000751 20421930 PMC2858681

[41] Jolliffe IT. Principal Component Analysis. 2nd ed New York: Springer; 2002.

[42] Mardia KV, Kent JT, Bibby JM. Multivariate Analysis. London: Academic Press; 1979.

[43] Guckenheimer J, Holmes P. Nonlinear Oscillations, Dynamical Systems, and Bifurcations of Vector Fields vol. 42 of Applied Mathematical Sciences. New York: Springer-Verlag; 1997.

[44] Hirsch MW, Smale S, Devaney RL. Differential Equations, Dynamical Systems and an Introduction to Chaos. 3rd ed Waltham, MA: Elsevier; 2004.

[45] Harris CM, Wolpert DM. Signal-Dependent Noise Determines Motor Planning. Nature. 1998;394(6695):780–784. 10.1038/29528 9723616

[46] van Beers RJ. Motor Learning Is Optimally Tuned to the Properties of Motor Noise. Neuron. 2009;63(3):406–417. 10.1016/j.neuron.2009.06.025 19679079

[47] Burge J, Ernst MO, Banks MS. The statistical determinants of adaptation rate in human reaching. Journal of Vision. 2008;8(4):1–19. 10.1167/8.4.20 18484859 PMC2684526

[48] Diedrichsen J, Hashambhoy Y, Rane T, Shadmehr R. Neural Correlates of Reach Errors. The Journal of Neuroscience. 2005;25(43):9919–9931. 10.1523/JNEUROSCI.1874-05.2005 16251440 PMC1479774

[49] Verhulst F. Nonlinear Differential Equations and Dynamical Systems 2nd ed Universitext. New York: Springer-Verlag; 1996.

[50] Khalil HK. Nonlinear systems. 3rd ed New Jersey: Prentice Hall; 2002.

[51] Rácz K, Valero-Cuevas F. Spatio-temporal analysis reveals active control of both task-relevant and task-irrelevant variables. Frontiers in Computational Neuroscience. 2013;7(155). 10.3389/fncom.2013.00155 24312045 PMC3826108

[52] Terrier P, Dériaz O. Persistent and anti-persistent pattern in stride-to-stride variability of treadmill walking: Influence of rhythmic auditory cueing. Human Movement Science. 2012;31(6):1585–1597. 10.1016/j.humov.2012.05.004 23164626

[53] Terrier P. Step-to-Step Variability in Treadmill Walking: Influence of Rhythmic Auditory Cueing. PLoS ONE. 2012;7(10):e47171 10.1371/journal.pone.0047171 23056604 PMC3466237

[54] van Beers RJ, Brenner E, Smeets JBJ. Random walk of motor planning in task-irrelevant dimensions. Journal of Neurophysiology. 2013;109(4):969–977. 10.1152/jn.00706.2012 23175799

[55] Hausdorff JM, Peng CK, Ladin Z, Wei JY, Goldberger AL. Is Walking a Random Walk? Evidence for Long-Range Correlations in Stride Interval of Human Gait. Journal of Applied Physiology. 1995;78(1):349–358. 7713836 10.1152/jappl.1995.78.1.349

[56] Peng CK, Buldyrev SV, Goldberger AL, Havlin S, Sciortino F, Simons M, et al Long-Range Correlations in Nucleotide Sequences. Nature. 1992;356(6365):168–170. 10.1038/356168a0 1301010

[57] Delignières D, Torre K. Fractal Dynamics of Human Gait: A Reassessment of the 1996 Data of Hausdorff et al. Journal of Applied Physiology. 2009;106(4):1272–1279. 10.1152/japplphysiol.90757.2008 19228991

[58] Maraun D, Rust HW, Timmer J. Tempting Long-Memory: On the Interpretation of DFA Results. Nonlinear Processes in Geophysics. 2004;11(4):495–503. 10.5194/npg-11-495-2004

[59] Gao J, Hu J, Tung WW, Cao Y, Sarshar N, Roychowdhury VP. Assessment of Long-Range Correlation in Time Series: How to Avoid Pitfalls. Physical Review E. 2006;73:016117 10.1103/PhysRevE.73.016117 16486226

[60] Marple SL Jr. Digital Spectral Analysis with Applications. Englewood Cliffs, NJ: Prentice Hall; 1987.

[61] Shumway RH, Stoffer DS. Time Series Analysis and Its Applications. New York: Springer; 2000.

[62] Kantz H, Schreiber T. Nonlinear Time Series Analysis. 2nd ed Cambridge, UK: Cambridge University Press; 2004.

[63] Kirchner M, Schubert P, Liebherr M, Haas CT. Detrended Fluctuation Analysis and Adaptive Fractal Analysis of Stride Time Data in Parkinson’s Disease: Stitching Together Short Gait Trials. PLoS ONE. 2014;9(1):e85787 10.1371/journal.pone.0085787 24465708 PMC3900445

[64] Peng CK, Havlin S, Stanley HE, Goldberger AL. Quantification of Scaling Exponents and Crossover Phenomena in Nonstationary Heartbeat Time Series. Chaos. 1995;5(1):82–87. 10.1063/1.166141 11538314

[65] Verrel J, Pradon D, Vuillerme N. Persistence of Motor-Equivalent Postural Fluctuations during Bipedal Quiet Standing. PLoS ONE. 2012;7(10):e48312 10.1371/journal.pone.0048312 23110228 PMC3482199

[66] van Beers RJ. How Does Our Motor System Determine Its Learning Rate? PLoS ONE. 2012;7(11):e49373 10.1371/journal.pone.0049373 23152899 PMC3495886

